# Systematic review and network meta-analysis of non-pharmacological interventions for inadequate perfusion in hypothermic circulatory arrest

**DOI:** 10.1016/j.isci.2026.116722

**Published:** 2026-07-17

**Authors:** Lingda Meng, Zhijing Wei, Xuan Jiang, Chunmao Lu, Qin Fang, Peng Yu, Zhiwei Zhang, Enyi Shi, Tianxiang Gu

**Affiliations:** 1 Department of Cardiac Surgery, First Affiliated Hospital, China Medical University, Shenyang, China; 2 Institute of Trauma Center, First Affiliated Hospital, China Medical University, Shenyang, China

**Keywords:** hypothermic circulatory arrest, non-pharmacological interventions, inadequate perfusion, network meta-analysis

## Abstract

This network meta-analysis aimed to compare different non-pharmacological interventions for insufficient perfusion after hypothermic circulatory arrest, a key technique in aortic arch surgery. The authors retrieved and analyzed 60 eligible studies from four databases up to July 2025. The results showed that moderate hypothermia combined with specific cerebral perfusion strategies achieved better overall outcomes. Moderate hypothermia generally improved multiple clinical indicators without affecting several complications, and different perfusion approaches and cannulation methods had comparable effects. This work helps surgeons select optimal interventions and improve patient prognosis after aortic arch surgery.

## Highlights

• MHCA improves outcomes for mortality, PTD/TND, renal failure/dialysis, and ventilation time
• RCP cannot improve outcomes related to inadequate perfusion when compared with ACP or BACP
• IA-ACP matches ACP/BACP outcomes in MHCA, an alternative to standard ACP
• MHCA combined with ACP or BACP yields better clinical outcomes in HCA patients

## Teaser

Health sciences; Medicine; Medical specialty; Internal medicine; Cardiovascular medicine

## Introduction

**Figure 1:**
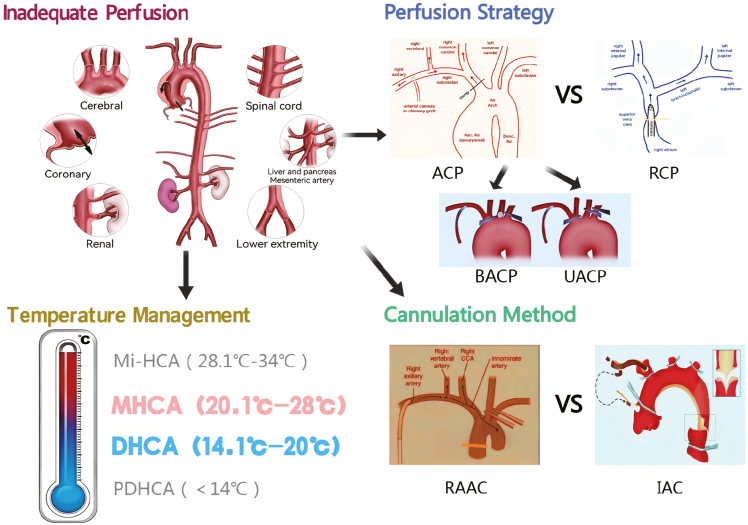
Non-pharmacological intervention and cerebral protection strategies for insufficient perfusion during hypothermic circulatory arrest This schematic diagram illustrates core non-pharmacological intervention and cerebral protection strategies targeting insufficient perfusion under hypothermic circulatory arrest. It comprehensively depicts diverse cerebral perfusion approaches, the principle of perfusion strategy selection, standardized hypothermic temperature management regimens, and key operational technical details of routine cerebral protection procedures.

Hypothermic circulatory arrest (HCA) is a component of the extracorporeal circulation process and a critical technique in cardiac surgery. By lowering the patient’s core temperature, it provides a bloodless and quiescent surgical field for complex aortic arch procedures, such as arch replacement and dissection repair.^1^ In 1975, Griepp successfully employed deep HCA (DHCA) in aortic arch replacement surgery.^2^ Subsequently, DHCA was widely adopted in various cardiac surgical procedures, leading to rapid advancements in the field. However, clinical studies have indicated that the isolated use of DHCA in aortic arch surgery may lead to inadequate perfusion, which can cause ischemia and hypoxia in various organs, triggering a cascade of changes that ultimately result in organ damage.^3^^,^^4^^,^^5^ Inadequate perfusion primarily includes cerebral hypoperfusion, as well as spinal cord and renal hypoperfusion. Cerebral inadequate perfusion is the most prevalent and detrimental, with numerous animal studies using clinical disease and injury models demonstrating brain injury resulting from HCA.^6^^,^^7^^,^^8^^,^^9^ Pharmacological interventions during HCA are limited, with some, such as the addition of nitric oxide during cardiopulmonary bypass, showing limited benefit.^10^ Furthermore, some studies have explored inflammation-related and hypoxia-related pathways, using pathway activators or inhibitors for intervention, while others have utilized bioinformatics approaches, such as genomics, proteomics, and metabolomics, for analysis. However, these interventions are largely confined to the preclinical stage, with limited clinical translation. Consequently, non-pharmacological interventions remain the primary focus of research on circulatory arrest. These non-pharmacological interventions can be broadly categorized into the following three areas: perfusion strategies, temperature management, and cannulation methods (Figure 1). Most current studies primarily focus on pairwise comparisons of specific interventions within the categories of perfusion strategies, temperature management, and cannulation methods, with few studies comprehensively comparing multiple non-pharmacological interventions.

Network meta-analysis is an extension of traditional meta-analysis that allows for the comprehensive comparison of multiple interventions, constructs complex network diagrams, and facilitates ranking, making it particularly suitable for clinical and evidence-based medicine. This study obtained data from randomized controlled trials (RCTs) and observational cohort studies and assessed the impact of HCA-related non-pharmacological interventions on inadequate perfusion outcomes. The aim of this network meta-analysis was to comparatively evaluate the efficacy of various non-pharmacological interventions and their combinations in improving inadequate perfusion-related outcomes, thereby enhancing the understanding of organ protection strategies, especially those focused on cerebral protection.

## Results

### Search results for included studies

**Figure 2:**
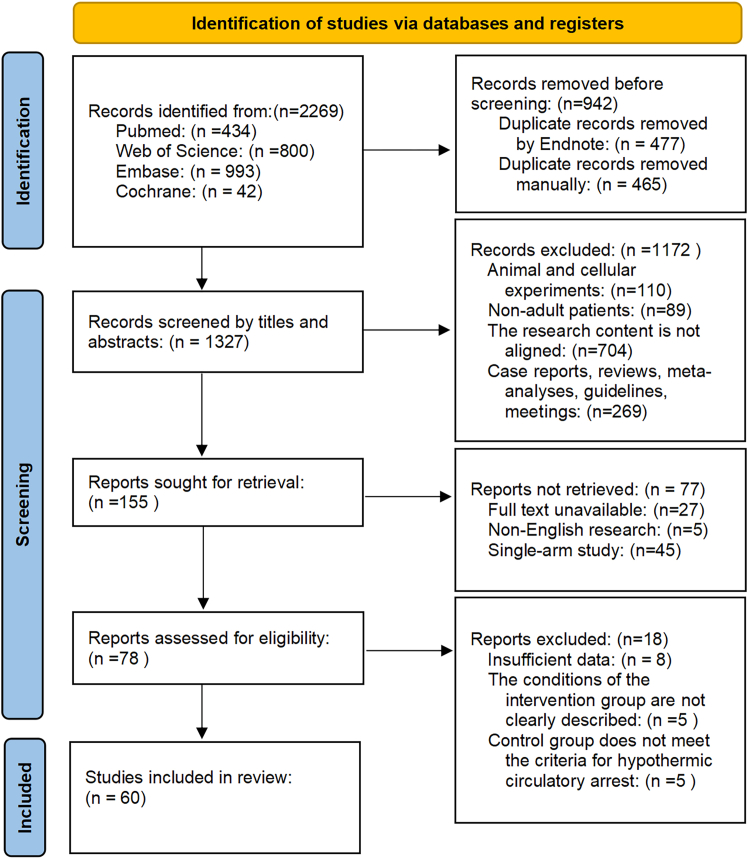
PRISMA 2020 flowchart of literature screening for the systematic review This flowchart presents the complete literature screening process following the PRISMA 2020 guidelines. It records the number of studies retrieved from databases and quantifies the included and excluded studies at each step of deduplication, title and abstract screening, and full-text assessment, with detailed exclusion reasons clarified for each screening stage.

A database search yielded 2,269 relevant studies, including Pubmed (*n* = 434), Web of Science (*n* = 800), Embase (*n* = 993), and Cochrane (*n* = 42). After removing duplicates (*n* = 942), 1,172 studies were excluded based on title and abstract review and 77 studies were excluded after full-text review. A total of 60 studies were ultimately included, comprising 4 RCTs and 56 observational cohort studies (OCS) studies. The process of study selection is detailed in Figure 2.

### Description of the included studies and the network

This network meta-analysis included 60 studies, encompassing a total of 22,095 patients. The basic information of the included studies is presented in Table 1. Nine non-pharmacological interventions were involved, and the number of studies for each intervention was as follows: DHCA (20), DHCA + antegrade cerebral perfusion (ACP) (25), DHCA + retrograde cerebral perfusion (RCP) (16), DHCA + bilateral anterograde cerebral perfusion (BACP) (5), moderate HCA (MHCA) (5), MHCA + ACP (41), MHCA + RCP (4), MHCA + BACP (9), and MHCA + innominate artery antegrade cerebral perfusion (IA-ACP) (4). The primary outcomes and the number of studies including these events were mortality (60), permanent neurological dysfunction (PND) (58), and transient neurological dysfunction (TND) (43). The secondary outcomes and the number of studies including these events were renal failure/dialysis (48), paraplegia (22), intensive care unit (ICU) time duration (38), and ventilation duration (32).

**Table 1:** Basic information of included studies

Author	Year	Journal	Country	Study type	Disease type	Surgical period	Intervention	Sample size
Huadong Li	2021	The Heart Surgery Forum	China	OCS	AD	2016–2018	6 vs. 8	88
Mark D. Peterson	2020	Journal of Thoracic and Cardiovascular Surgery	Canada	RCT	AA	2015–2018	6 vs. 9	111
George Matalanis	2003	Annals of Thoracic and Cardiovascular Surgery	Australia	OCS	AA and AD	1996–2000	1 vs. 2 vs. 3	62
Christian Olsson	2006	Annals of Thoracic Surgery	Sweden	OCS	AA and AD	2001–2004	2 vs. 4	34
Sergey Leontyev	2019	European Journal of Cardio-Thoracic Surgery	Germany	OCS	AA	1995–2014	5 vs. 8	420
Efstratios Apostolakis	2008	Journal of Cardiac Surgery	Greece	OCS	AD	1998–2006	2 vs. 3	48
Elizabeth L. Norton	2022	JTCVS Techniques	USA	OCS	AD	2015–2020	6 vs. 9	264
YanWenWu	2017	Perfusion	China	OCS	AA and AD	2013–2014	2 vs. 6	109
Richard C. Cook	2006	Journal of Cardiac Surgery	Canada	OCS	AA and AD	1995–2002	2 vs. 6	68
Yuji Miyamoto	2003	Annals of Thoracic and Cardiovascular Surgery	Japan	OCS	AA and AD	1993–2001	1 vs. 2	23
Shuyang Lu	2017	Annals of Thoracic Surgery	China	OCS	AA and AD	2005–2011	2 vs. 4	263
Guang Tong	2017	Journal of Thoracic and Cardiovascular Surgery	China	OCS	AA	2006–2014	6 vs. 8	203
Yoshiyuki Tokuda	2014	Circulation Journal	Japan	OCS	AD	2008–2012	6 vs. 7	2,640
Tobias Krüger	2011	Circulation	Germany	OCS	AD	2006–2009	1 vs. 2 vs. 4	1,436
George J. Arnaoutakis	2022	Journal of Cardiac Surgery	USA	OCS	AA	2014–2020	1 vs. 3	320
Wenhui Zhang	2025	Current Problems in Surgery	China	OCS	AD	2019–2023	6 vs. 8	156
Anna K. Gergen	2023	Journal of Surgical Research	USA	OCS	AA and AD	2010–2021	6 vs. 7	319
Peiqing Dong	2002	Journal of Extra-Corporeal Technology	China	OCS	AA	1985–2000	1 vs. 3	65
Kai Zhu	2022	Annals of Translational Medicine	China	OCS	AA	2017–2021	2 vs. 6 vs. 8	138
Marco Di Eusanio	2003	Journal of Thoracic and Cardiovascular Surgery	Netherlands	OCS	AA and AD	1995–2021	1 vs. 6	289
Oksana Vasilyevna Kamenskaya	2017	Journal of Extra-Corporeal Technology	Russia	RCT	AD	2011–2012	1 vs. 6	58
Song-Bo Dong	2020	Journal of Cardiothoracic Surgery	China	OCS	AD	2019–2025	6 vs. 8	61
A. Faruk Hokenek	2016	Journal of Thoracic and Cardiovascular Surgery	Turkey	OCS	AA and AD	2007–2012	6 vs. 9	69
Marion Mauduit	2021	Journal of Cardiovascular Medicine	France	OCS	AA and AD	1995–2016	1 vs. 6	424
Dominik Wiedemann	2013	Journal of Thoracic and Cardiovascular Surgery	Austria	OCS	AD	1987–2011	1 vs. 3 vs. 6	329
R. S. Bonser	2002	Journal of Thoracic and Cardiovascular Surgery	UK	RCT	AA and AD	1998–1999	1 vs. 3	42
Wei Wang	2025	International Journal of Cardiology	China	OCS	AD	2013–2021	2 vs. 6	817
Andreas Zierer	2007	Annals of Thoracic Surgery	USA	OCS	AD	1984–2005	1 vs. 3	125
Zhongrong Fang	2019	Interactive Cardiovascular and Thoracic Surgery	China	OCS	AD	2013–2016	2 vs. 6	434
Yingjie Du	2021	Frontiers in Surgery	China	OCS	AD	2013–2016	2 vs. 6	382
Ming Gong	2016	Journal of Thoracic Disease	China	OCS	AD	2014–2015	2 vs. 6	74
Mingjia Ma	2016	Thoracic and Cardiovascular Surgeon	China	OCS	AD	2010–2013	2 vs. 6	99
Stevan S. Pupovac	2021	Annals of Thoracic Surgery	USA	OCS	AD	2010–2018	1 vs. 6	724
Bradley G. Leshnower	2015	Annals of Thoracic Surgery	USA	OCS	AD	2004–2014	2 vs. 6	288
Prashanth Vallabhajosyula	2015	Annals of Thoracic Surgery	USA	OCS	AA and AD	2008–2012	3 vs. 9	376
Hong Qian	2013	Journal of Cardiothoracic Surgery	China	OCS	AD	2007–2012	2 vs. 6	54
Umberto Benedetto	2021	European Journal of Cardio-Thoracic Surgery	UK	OCS	AD	2011–2018	1 vs. 2 vs. 3 vs. 4	1,929
Andrei M. Belyaev	2024	Journal of Surgical Research	Russia	OCS	AD	2003–2023	2 vs. 6	549
Khaled D. Algarni	2014	Journal of Thoracic and Cardiovascular Surgery	Canada	OCS	AD	1990–2010	3 vs. 7	128
Yutaka Okita	2001	Annals of Thoracic Surgery	Japan	RCT	AA	1997–2020	3 vs. 6	60
Hiroyuki Kamiya	2007	Annals of Thoracic Surgery	Germany	OCS	AA	2000–2005	5 vs. 6	61
James J. Livesay	1983	Annals of Thoracic Surgery	USA	OCS	AA and AD	1976–0982	1 vs. 5	60
Rita Karianna Milewski	2010	Annals of Thoracic Surgery	Italy	OCS	AA and AD	1997–2008	3 vs. 6	776
William B. Keeling	2023	Innovations	USA	OCS	AA and AD	2007–2012	3 vs. 6	300
Jacob Ede	2024	Journal of Cardiothoracic Surgery	Sweden	OCS	AD	1998–2022	1 vs. 3	515
Sotiris C. Stamou	2018	The International Journal of Angiology	USA	OCS	AD	2000–2014	1 vs. 5	132
W. Brent Keeling	2018	Annals of Thoracic Surgery	USA	OCS	AA and AD	2000–2015	2 vs. 6	1,338
D.K. Harrington	2004	Circulation	UK	RCT	AA and AD	2001–2003	1 vs. 6	42
Mohammad Shihata	2011	Journal of Thoracic and Cardiovascular Surgery	Canada	OCS	AA and AD	2001–2009	1 vs. 6	124
Michael E. Halkos	2009	Journal of Thoracic and Cardiovascular Surgery	USA	OCS	AA and AD	2004–2007	1 vs. 6	271
Hyuk Ahn	1997	Cardiovascular Surgery	Korea	OCS	AD	1986–1994	1 vs. 2 vs. 3	47
Hend Abdulwahab	2024	Frontiers in Cardiovascular Medicine	Switzerland	OCS	AD	2011–2020	2 vs. 6	143
George J. Arnaoutakis	2016	Annals of Thoracic Surgery	USA	OCS	AA and AD	2009–2014	3 vs. 6	586
Bowen Li	2017	International Journal of Surgery	China	OCS	AA and AD	2013–2015	2 vs. 4	77
Kai Zhang	2024	European Journal of Cardio-Thoracic Surgery	China	OCS	AD	2010–2018	2 vs. 6	657
Hiroyuki Kamiya	2007	Journal of Thoracic and Cardiovascular Surgery	Germany	OCS	AA and AD	1999–2005	2 vs. 6	184
Andreas Zierer, MD	2013	Thoracic and Cardiovascular Surgeon	Germany	OCS	AA	2000–2012	6 vs. 8	492
Antonio Piperata	2022	European Journal of Cardio-Thoracic Surgery	Italy	OCS	AD	2008–2018	6 vs. 8	378
Martin Misfeld	2012	Annals of Thoracic Surgery	Germany	OCS	AA and AD	2003–2009	5 vs. 6 vs. 7 vs. 8	636

AA, aortic aneurysm; AD, aortic dissection.

### Results of the quality assessment and risk-of-bias evaluation of the included studies

**Figure 3:**
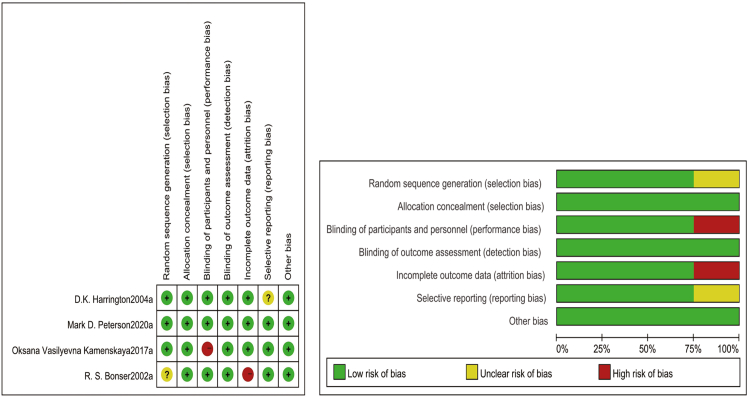
Risk-of-bias assessment of included studies This bar chart evaluates the risk of bias across multiple dimensions for all eligible included studies, covering selection bias, performance bias, follow-up bias, and reporting bias. Bias levels are intuitively classified as low, moderate, and high risk, marked by green, yellow, and red color coding, respectively.

The Cochrane Risk of Bias tool (RoB 2) was used to assess the included RCTs, and the quality assessment and risk-of-bias analysis are presented in Figure 3.

This network meta-analysis included both RCTs and observational cohort studies; therefore, we could not determine the exact quality levels of evidence for the outcomes. We provided details of the influencing factors.

The Newcastle-Ottawa quality assessment scale (NOS) was used to assess the quality of observational cohort studies. We evaluated each item of the NOS for all observational cohort studies, calculated the total scores, and summarized the results, as shown in Table 2.

**Table 2:** Quality assessment of observational cohort studies

Number	Author	Year	Assessment of outcome	Selection of the non-exposed cohort	Ascertainment of exposure	Demonstration that outcome of interest was not present at start of study	Comparability of cohorts on the basis of the design or analysis controlled for confounders	Assessment of outcome	Was follow-up long enough for outcomes to occur	Adequacy of follow-up of cohorts	Total score
1	Huadong Li	2021	1	1	1	1	2	1	1	0	8
2	George Matalanis	2003	0	1	1	1	2	1	1	0	7
3	Christian Olsson	2006	1	1	1	1	2	1	1	0	8
4	Sergey Leontyev	2019	1	1	1	1	2	1	1	0	8
5	Efstratios Apostolakis	2008	1	1	1	1	1	1	1	0	7
6	YanWenWu	2017	1	1	1	1	2	1	1	0	8
7	Richard C. Cook	2006	1	1	1	1	2	1	1	0	8
8	Yuji Miyamoto	2003	1	1	1	1	1	1	1	0	7
9	Shuyang Lu	2017	1	1	1	1	1	1	1	0	8
10	Guang Tong	2017	1	1	1	1	1	1	1	0	8
11	Yoshiyuki Tokuda	2014	1	1	1	1	1	1	1	0	7
12	Tobias Krüger	2011	1	1	1	1	2	1	1	1	9
13	George J. Arnaoutakis	2022	1	1	1	1	1	1	1	0	7
14	Wenhui Zhang	2025	1	1	1	1	2	1	1	0	8
15	Anna K. Gergen	2023	1	1	1	1	1	1	1	0	7
16	Peiqing Dong	2002	1	1	1	1	1	1	1	0	7
17	Kai Zhu	2022	1	1	1	1	1	1	1	1	8
18	Marco Di Eusanio	2003	1	1	1	1	2	1	1	0	8
19	Song-Bo Dong	2020	1	1	1	1	2	1	1	1	9
20	A. Faruk Hokenek	2016	1	1	1	1	1	1	1	0	7
21	Xiaomeng Wang	2025	1	1	1	1	2	1	1	0	8
22	Marion Mauduit	2021	1	1	1	1	1	1	1	0	7
23	Dominik Wiedemann	2013	1	1	1	1	2	1	1	0	8
24	Wei Wang	2025	1	1	1	1	2	1	1	0	8
25	Andreas Zierer	2007	1	1	0	1	1	1	1	0	6
26	Zhongrong Fang	2019	1	1	1	1	1	1	1	1	8
27	Yingjie Du	2021	1	1	1	1	2	1	1	0	8
28	Ming Gong	2016	1	1	1	1	1	1	1	0	7
29	Mingjia Ma	2016	1	1	1	1	1	1	1	1	8
30	Stevan S. Pupovac	2021	1	1	1	1	1	1	1	1	8
31	Bradley G. Leshnower	2015	1	1	1	1	2	1	1	0	8
32	Prashanth Vallabhajosyula	2015	1	1	1	1	2	1	1	1	9
33	Hong Qian	2013	1	1	1	1	1	1	1	1	8
34	Umberto Benedetto	2021	1	1	1	1	1	1	1	1	9
35	Andrei M. Belyaev	2024	1	1	1	1	1	1	1	1	8
36	Khaled D. Algarni	2014	1	1	1	1	1	1	1	1	8
37	Yutaka Okita	2001	1	1	1	1	1	0	1	1	7
38	Hiroyuki Kamiya	2007	1	1	1	1	2	1	1	0	8
39	James J. Livesay	1983	1	1	1	1	1	1	1	0	7
40	Rita Karianna Milewski	2010	1	1	0	1	2	1	1	1	8
41	William B. Keeling	2023	1	1	1	1	1	1	1	1	8
42	Jacob Ede	2024	1	1	1	1	2	1	1	1	9
43	Sotiris C. Stamou	2018	1	1	0	1	1	1	1	1	7
44	W. Brent Keeling	2018	1	1	1	1	2	1	1	1	9
45	Mohammad Shihata	2011	1	1	1	1	1	1	1	1	8
46	Michael E. Halkos	2009	1	1	1	1	2	1	1	1	9
47	Hyuk Ahn	1997	1	1	1	1	2	1	1	0	8
48	Hend Abdulwahab Muftah Abdulwahab	2024	1	1	1	1	2	1	1	1	9
49	George J. Arnaoutakis	2016	1	1	1	1	2	1	1	0	8
50	Bowen Li	2017	1	1	1	1	2	1	1	1	9
51	Kai Zhang	2024	1	1	1	1	2	1	1	1	9
52	Hiroyuki Kamiya	2007	1	1	1	1	1	1	1	0	7
53	Andreas Zierer, MD	2013	1	1	1	1	2	1	1	1	9
54	Antonio Piperata	2022	1	1	1	1	2	1	1	0	8
55	Martin Misfeld	2012	1	1	1	1	1	1	1	1	8
56	Elizabeth L. Norton	2022	1	1	0	1	1	1	1	1	7

The Risk of Bias in Non-Randomized Studies of Interventions (ROBINS-I) was used to assess the risk of bias of observational cohort studies. We evaluated each domain of the ROBINS-I for all observational cohort studies, and the results are summarized in Table 3.

**Table 3:** Risk-of-bias assessment in observational cohort studies by ROBINS-I

Number	Author	Year	Bias due to confounding	Bias in selection of participants into the study	Bias in classification of interventions	Bias due to deviations from intended	Bias due to missing data	Bias in measurement of outcomes	Bias in selection of the reported result	Overall
1	Huadong Li	2021	Serious	Low	Low	Low	Low	Low	Low	Serious
2	George Matalanis	2003	Low	Low	Moderate	Low	Low	Low	Low	Moderate
3	Christian Olsson	2006	Moderate	Low	Low	Low	Low	Low	Low	Moderate
4	Sergey Leontyev	2019	Serious	Low	Low	Low	Low	Low	Low	Serious
5	Efstratios Apostolakis	2008	Moderate	Low	Low	Low	Low	Low	Low	Moderate
6	YanWenWu	2017	Low	Low	Low	Low	Low	Low	Low	Low
7	Richard C. Cook	2006	Moderate	Low	Low	Low	Low	Low	Low	Moderate
8	Yuji Miyamoto	2003	Serious	Low	Low	Low	Moderate	Low	Low	Serious
9	Shuyang Lu	2017	Moderate	Low	Low	Low	Moderate	Low	Low	Moderate
10	Guang Tong	2017	Low	Low	Low	Low	Low	Low	Low	Low
11	Yoshiyuki Tokuda	2014	Serious	Low	Low	Low	Moderate	Low	Low	Serious
12	Tobias Krüger	2011	Low	Low	Low	Low	Low	Low	Low	Low
13	George J. Arnaoutakis	2022	Moderate	Low	Low	Low	Moderate	Low	Low	Moderate
14	Wenhui Zhang	2025	Moderate	Low	Low	Low	Low	Low	Low	Moderate
15	Anna K. Gergen	2023	Serious	Low	Low	Low	Low	Low	Low	Serious
16	Peiqing Dong	2002	Moderate	Low	Low	Low	Low	Low	Low	Moderate
17	Kai Zhu	2022	Low	Low	Low	Low	Low	Low	Low	Low
18	Marco Di Eusanio	2003	Low	Low	Low	Low	Low	Low	Low	Low
19	Song-Bo Dong	2020	Low	Low	Low	Low	Low	Low	Low	Low
20	A. Faruk Hokenek	2016	Moderate	Low	Low	Low	Low	Low	Low	Moderate
21	Xiaomeng Wang	2025	Low	Low	Low	Low	Low	Low	Low	Low
22	Marion Mauduit	2021	Serious	Low	Low	Moderate	Low	Low	Low	Serious
23	Dominik Wiedemann	2013	Moderate	Low	Low	Low	Moderate	Low	Low	Moderate
24	Wei Wang	2025	Serious	Low	Low	Low	Moderate	Low	Low	Serious
25	Andreas Zierer	2007	Moderate	Low	Low	Low	Moderate	Low	Low	Moderate
26	Zhongrong Fang	2019	Moderate	Low	Low	Low	Low	Low	Low	Moderate
27	Yingjie Du	2021	Low	Low	Low	Low	Low	Low	Low	Low
28	Ming Gong	2016	Low	Low	Low	Low	Low	Low	Low	Low
29	Mingjia Ma	2016	Low	Low	Low	Low	Low	Low	Low	Low
30	Stevan S. Pupovac	2021	Moderate	Moderate	Low	Low	Low	Low	Low	Moderate
31	Bradley G. Leshnower	2015	Moderate	Low	Low	Low	Low	Low	Low	Moderate
32	Prashanth Vallabhajosyula	2015	Low	Low	Low	Low	Low	Low	Low	Low
33	Hong Qian	2013	Low	Low	Low	Low	Low	Low	Low	Low
34	Umberto Benedetto	2021	Moderate	Low	Low	Low	Low	Low	Low	Moderate
35	Andrei M. Belyaev	2024	Low	Low	Low	Low	Low	Low	Low	Low
36	Khaled D. Algarni	2014	Moderate	Low	Low	Low	Low	Low	Low	Moderate
37	Yutaka Okita	2001	Low	Low	Low	Low	Moderate	Moderate	Low	Moderate
38	Hiroyuki Kamiya	2007	Low	Low	Low	Low	Low	Low	Low	Low
39	James J. Livesay	1983	Moderate	Low	Moderate	Low	Low	Low	Low	Moderate
40	Rita Karianna Milewski	2010	Moderate	Low	Low	Low	Low	Low	Low	Moderate
41	William B. Keeling	2023	Low	Low	Low	Low	Low	Low	Low	Low
42	Jacob Ede	2024	Low	Low	Low	Low	Low	Low	Low	Low
43	Sotiris C. Stamou	2018	Serious	Low	Low	Low	Low	Low	Low	Serious
44	W. Brent Keeling	2018	Low	Low	Low	Low	Low	Low	Low	Low
45	Mohammad Shihata	2011	Serious	Low	Low	Low	Low	Low	Low	Serious
46	Michael E. Halkos	2009	Low	Low	Low	Low	Low	Low	Low	Low
47	Hyuk Ahn	1997	Moderate	Low	Low	Low	Low	Low	Low	Moderate
48	Hend Abdulwahab	2024	Low	Low	Low	Low	Low	Low	Low	Low
49	George J. Arnaoutakis	2016	Moderate	Low	Low	Low	Low	Low	Low	Moderate
50	Bowen Li	2017	Low	Low	Low	Low	Low	Low	Low	Low
51	Kai Zhang	2024	Low	Low	Low	Low	Low	Low	Low	Low
52	Hiroyuki Kamiya	2007	Serious	Low	Low	Moderate	Low	Low	Low	Serious
53	Andreas Zierer, MD	2013	Moderate	Low	Low	Low	Low	Low	Low	Moderate
54	Antonio Piperata	2022	Low	Low	Low	Low	Low	Low	Low	Low
55	Martin Misfeld	2012	Low	Low	Low	Low	Low	Low	Low	Low
56	Elizabeth L. Norton	2022	Moderate	Low	Low	Low	Low	Low	Low	Moderate

**Figure 4:**
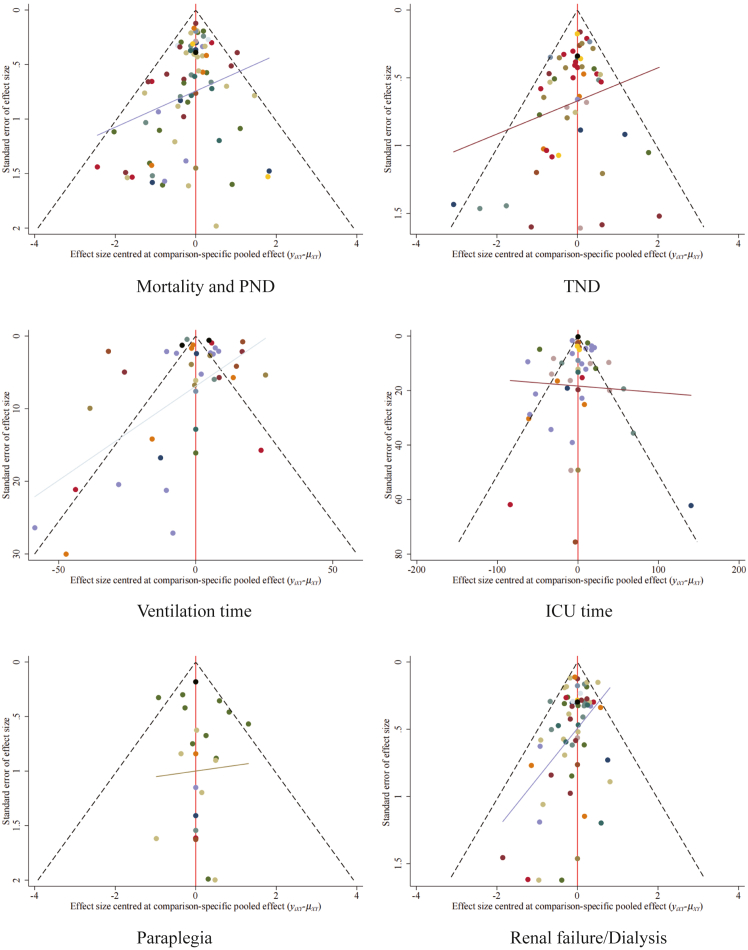
Distribution and correlation of clinical indicators under different non-pharmacological interventions This scatterplot consists of multiple subgraphs to visualize the distribution characteristics of clinical indicators and the correlation between variables corresponding to different non-pharmacological intervention strategies.

Publication bias was evaluated using Egger’s test, and funnel plots were generated for visual inspection (Figure 4).

### Network evidence plot and inconsistency checks

**Figure 5:**
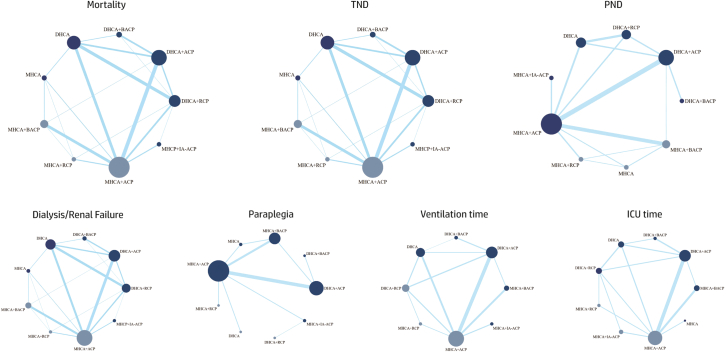
Network analysis of included non-pharmacological interventions This network diagram visualizes the correlational relationships among all non-pharmacological interventions. Nodes represent individual intervention measures, and connecting lines indicate pairwise comparative studies between different interventions.

This network meta-analysis provided evidence network diagrams for seven outcomes, as illustrated in Figure 5. Each network diagram encompassed various direct and indirect comparisons among nine non-pharmacological interventions. The width of the lines corresponded to the number of studies associated with each comparison, while the size of the circles reflected the sample size of each intervention group.

We performed inconsistency tests on seven outcomes. No significant inconsistencies were found for any of the indicators except renal failure/dialysis (*p* = 0.038). For the renal failure/dialysis indicator, local inconsistency tests revealed inconsistencies in DHCA + RCP vs. MHCA + RCP (*p* = 0.045), DHCA vs. MHCA (*p* = 0.045), and MHCA vs. MHCA + RCP (*p* = 0.035).

### Quality of evidence and rating of recommendations

The quality of evidence for each outcome was evaluated using the CINeMA framework integrated with the GRADE approach.^11^^,^^12^ The CINeMA framework, which is developed based on the GRADE system and specifically designed for network meta-analyses, assesses evidence quality across the following six core domains: within-study bias, reporting bias, indirectness, imprecision, heterogeneity, and incoherence. Each domain was judged as “no concern,” “some concern,” or “major concern.” The overall confidence in the estimated effect was then classified into the following four levels: high confidence, moderate confidence, low confidence, and very low confidence.

### Synthesize effect size analyses and rank-order findings

**Figure 6:**
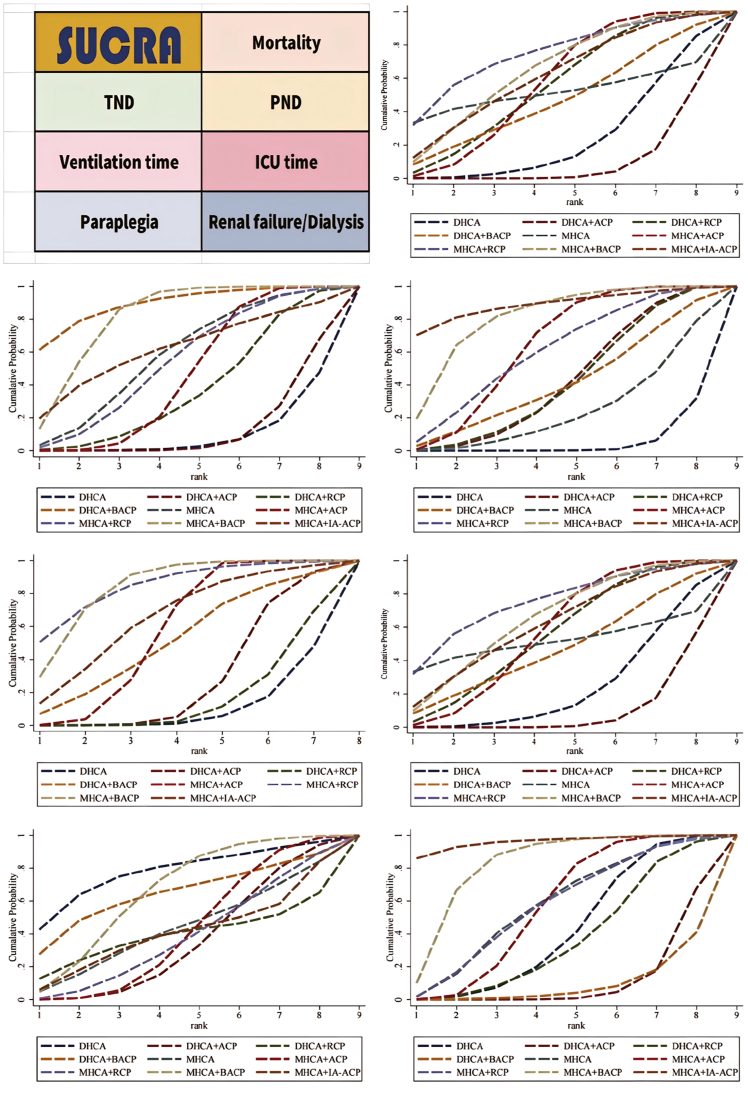
SUCRA ranking of non-pharmacological interventions for multiple clinical outcomes The SUCRA analysis ranks the efficacy of different non-pharmacological interventions based on key clinical outcomes, including mortality, neurological function recovery, ventilation time, ICU stay duration, and postoperative complication incidence.

This study employed a network meta-analysis to synthesize the comparative effects of nine non-pharmacological interventions on inadequate perfusion outcomes. Based on the surface under the cumulative ranking curve (SUCRA), cumulative probability curves were generated (Figure 6). The probability graphs and summaries for each non-pharmacological intervention are presented in Table 4.

**Table 4:** Ranking of intervention methods

Interventions	SUCRA (%) (mortality)	Ranking (mortality)	SUCRA (%) (PND)	Ranking (PND)	SUCRA (%) (TND)	Ranking (TND)	SUCRA (%) (ventilation time)	Ranking (ventilation time)	SUCRA (%) (ICU time)	Ranking (ICU time)	SUCRA (%) (paraplegia)	Ranking (paraplegia)	SUCRA (%) (renal failure/dialysis)	Ranking (renal failure/dialysis)
DHCA	18.9	9	4.9	9	9.7	9	10.3	9	24.3	8	78.1	1	42.8	6
DHCA + ACP	27.5	8	42.6	5	13.0	8	28.6	7	9.9	9	35.8	9	11.4	8
DHCA + RCP	34.3	6	42.0	6	37.3	7	16.4	8	56.0	5	38.7	8	36.7	7
DHCA + BACP	33.3	7	41.4	7	89.0	1	52.2	5	47.5	7	64.1	3	9.6	9
MHCA	61.2	4	24.6	8	58.0	4	57.6	4	51.8	6	44.1	4	57.4	3
MHCA + ACP	70.2	3	63.9	3	45.8	6	84.9	1	57.7	4	41.6	6	57.0	4
MHCA + RCP	84.1	1	60.8	4	54.2	5	84.1	2	75.1	1	39.3	7	57.0	4
MHCA + BACP	81.0	2	80.9	2	81.1	2	65.8	3	65.6	2	66.0	2	81.8	2
MHCA + IA-ACP	39.6	5	88.8	1	61.9	3	50.1	6	62.1	3	42.2	5	96.2	1

The three most effective interventions for reducing mortality were, in order, MHCA + RCP (SCURA: 84.1%), MHCA + BACP (SCURA: 81.0%), and MHCA +ACP (SCURA: 70.2%). The three most effective interventions for reducing PND incidence were, in order, MHCA + IA-ACP (SCURA: 88.8%), MHCA + BACP (SCURA: 80.9%), and MHCA + ACP (SCURA: 63.9%). The three most effective interventions for reducing TND incidence were, in order, DHCA + BACP (SCURA: 89.0%), MHCA + BACP (SCURA: 81.1%), and MHCA + IA-ACP (SCURA: 61.9%). The three most effective interventions for reducing ventilation time were, in order, MHCA + ACP (SCURA: 84.9%), MHCA + RCP (SCURA: 84.1%), and MHCA + BACP (SCURA: 65.8%). The three most effective interventions for reducing ICU time were, in order, MHCA + RCP (SCURA: 75.1%), MHCA + BACP (SCURA: 65.6%), and MHCA + IA-ACP (SCURA: 62.1%). The three most effective interventions for reducing paraplegia incidence are, in order, DHCA (SCURA: 78.1%), MHCA + BACP (SCURA: 66.0%), and DHCA + BACP (SCURA: 64.1%). The three most effective interventions for reducing renal failure/dialysis are, in order, MHCA + IA-ACP (SCURA: 96.2%), MHCA + BACP (SCURA: 81.8%), and MHCA (SCURA: 57.4%).

**Figure 7:**
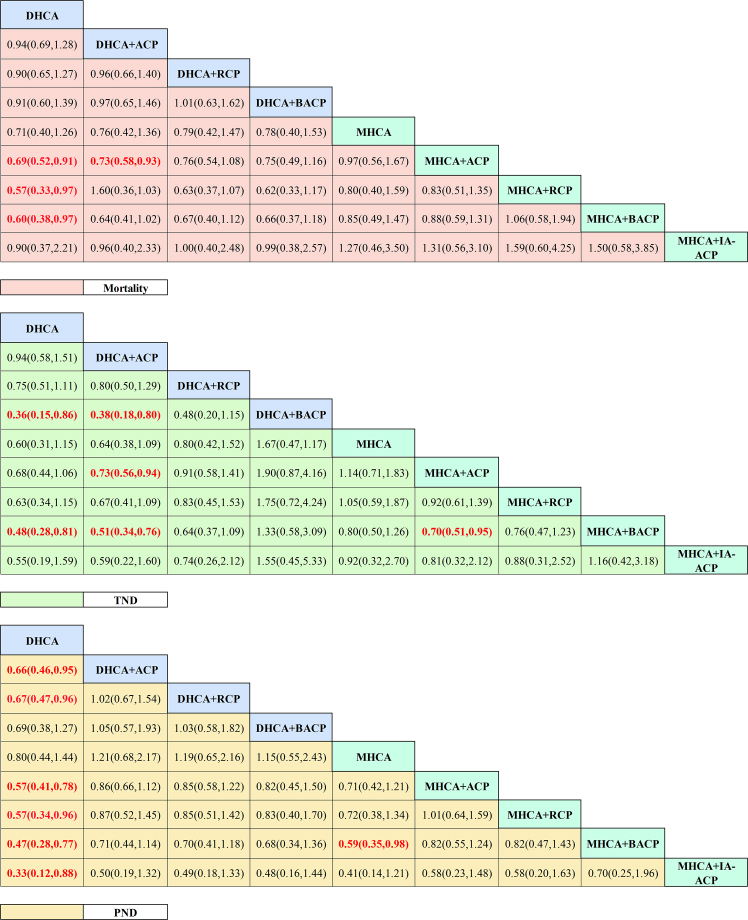
Pairwise comparison of primary outcomes among different non-pharmacological interventions This league table summarizes pairwise comparisons of primary clinical outcomes across all non-pharmacological interventions. Pooled effect sizes and 95% confidence intervals are presented to quantify the relative effects and statistical differences between interventions. Data are represented as mean ± SEM.

**Figure 8:**
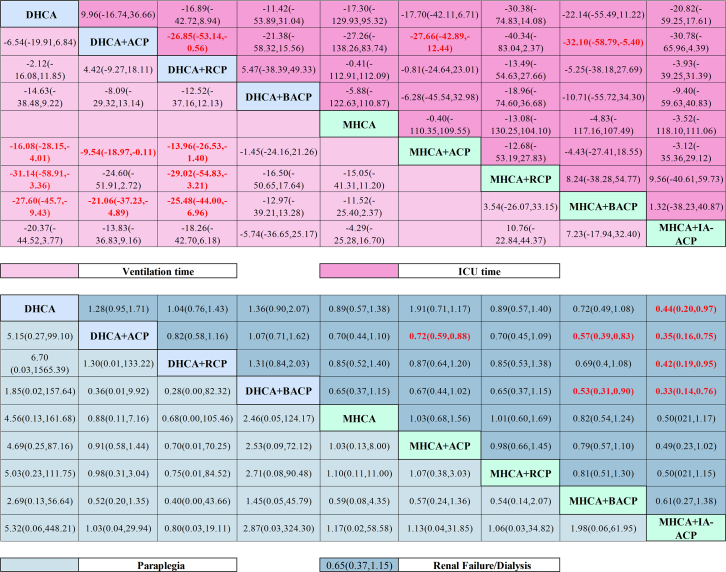
Pairwise comparison of secondary outcomes among different non-pharmacological interventions This league table exhibits pairwise comparative results of secondary outcomes, including ventilation time, ICU stay, paraplegia, and renal failure. Pooled effect values and statistical significance of each intervention comparison are fully demonstrated. Data are represented as mean ± SEM.

Figure 7 presents pairwise comparisons of the three primary outcomes: mortality, PND, and TND. Figure 8 presents pairwise comparisons of the four secondary outcomes: ventilation duration, ICU stay duration, paraplegia, and renal failure/dialysis. In this meta-analysis, the DHCA group served as the reference group, positioned in the upper-left corner of the league table.

In terms of mortality, statistically significant pairwise comparisons revealed differences between MHCA + ACP vs. DHCA (odds ratio [OR]: 0.69; 95% confidence interval [CI]: 0.52–0.91), MHCA + RCP vs. DHCA (OR: 0.57; 95% CI: 0.33–0.97), MHCA + ACP vs. DHCA (OR: 0.60; 95% CI: 0.38–0.97), and MHCA + ACP vs. DHCA + ACP (OR: 0.73; 95% CI: 0.58–0.93). These findings suggested that, compared to DHCA alone, MHCA combined with ACP, RCP, and bilateral cerebral perfusion could reduce mortality rates. Furthermore, MHCA + ACP was associated with lower mortality compared to DHCA + ACP.

In terms of PND, statistically significant pairwise comparisons revealed differences between DHCA + ACP vs. DHCA (OR: 0.66; 95% CI: 0.46–0.95), DHCA + RCP vs. DHCA (OR: 0.67; 95% CI: 0.47–0.96), MHCA + ACP vs. DHCA (OR: 0.57; 95% CI: 0.41–0.78), MHCA + RCP vs. DHCA (OR: 0.57; 95% CI: 0.34–0.96), MHCA + BACP vs. DHCA (OR: 0.47; 95% CI: 0.28–0.77), MHCA + IA-ACP vs. DHCA (OR: 0.33; 95% CI: 0.12–0.88), and MHCA + BACP vs. MHCA (OR: 0.73; 95% CI: 0.58–0.93). These findings suggested that, compared to DHCA alone, ACP, bilateral cerebral perfusion, ACP through innominate artery, and moderate hypothermia could reduce the incidence of PND.

In terms of TND, statistically significant pairwise comparisons revealed differences between DHCA + BACP vs. DHCA (OR: 0.36; 95% CI: 0.15–0.86), MHCA + BACP vs. DHCA (OR: 0.48; 95% CI: 0.28–0.81), DHCA + BACP vs. DHCA + ACP (OR: 0.38; 95% CI: 0.18–0.80), MHCA + ACP vs. DHCA + ACP (OR: 0.73; 95% CI: 0.56–0.94), MHCA + BACP vs. DHCA + ACP (OR: 0.51; 95% CI: 0.34–0.76), and MHCA + BACP vs. MHCA + ACP (OR: 0.70; 95% CI: 0.51–0.95). These findings suggested that, compared to DHCA alone, unilateral ACP, bilateral cerebral perfusion, and moderate hypothermia could reduce the incidence of TND. Compared to unilateral cerebral perfusion, bilateral cerebral perfusion demonstrated a superior advantage in reducing the incidence of TND under conditions of moderate hypothermia.

In terms of ventilation time, statistically significant pairwise comparisons revealed differences between MHCA + ACP vs. DHCA (mean difference [MD]: −16.08; 95% CI: −28.15, −4.01), MHCA + RCP vs. DHCA (MD: −31.14; 95% CI: −58.91, −3.36), MHCA + BACP vs. DHCA (MD: −27.60; 95% CI: −45.7, −9.43), MHCA + ACP vs. DHCA + ACP (MD: −9.54; 95% CI: −18.97, −0.11), MHCA + BACP vs. DHCA + ACP (MD: −21.06; 95% CI: −37.23, −4.89), MHCA + ACP vs. DHCA + RCP (MD: −13.96; 95% CI: −26.53, −1.40), and MHCA + BACP vs. DHCA + RCP (MD: −25.48; 95% CI: −44.00, −6.96). These findings suggested that, under deep hypothermic conditions, the application of ACP, RCP, or BACP did not reduce the ventilation duration in patients. However, under moderate hypothermic conditions, the application of ACP, RCP, or BACP could potentially decrease the ventilation duration in patients.

In terms of ICU time, statistically significant pairwise comparisons revealed differences between DHCA + RCP vs. DHCA + ACP (MD: −26.85; 95% CI: −53.14, −0.56), MHCA + ACP vs. DHCA + ACP (MD: −27.66; 95% CI: −42.89, −12.44), and MHCA + BACP vs. DHCA + ACP (MD: −32.10; 95% CI: −58.79, −5.40). These findings suggested that, compared to DHCA with ACP, MHCA with unilateral or bilateral ACP might reduce ICU time. However, the comparisons between other interventions were not statistically significant, indicating that interventions based on perfusion strategies, temperature management, and cannulation methods have a limited impact on reducing ICU time and are not the primary factors influencing ICU time.

In terms of paraplegia, no statistically significant differences were observed in any pairwise comparisons. These findings suggested that interventions based on perfusion strategies, temperature management, and cannulation techniques were not the primary determinants of postoperative paraplegia incidence.

In terms of renal failure/dialysis, statistically significant pairwise comparisons revealed differences between MHCA + IA-ACP vs. DHCA (OR: 0.44; 95% CI: 0.20–0.97), MHCA + ACP vs. DHCA + ACP (OR: 0.72; 95% CI: 0.59–0.88), MHCA + BACP vs. DHCA + ACP (OR: 0.57; 95% CI: 0.39–0.83), MHCA + IA-ACP vs. DHCA + ACP (OR: 0.35; 95% CI: 0.16–0.75), MHCA + IA-ACP vs. DHCA + RCP (OR: 0.42; 95% CI: 0.19–0.95), MHCA + BACP vs. DHCA + BACP (OR: 0.53; 95% CI: 0.31–0.90), and MHCA + IA-ACP vs. DHCA + BACP (OR: 0.33; 95% CI: 0.14–0.76). These findings suggested that, under deep hypothermic conditions, the utilization of ACP, RCP, or BACP might not mitigate the incidence of renal failure/dialysis in patients. Conversely, the utilization of ACP, BACP, or IA-ACP under moderate hypothermic conditions might potentially decrease the incidence of renal failure/dialysis. Furthermore, IA-ACP might be superior in reducing the incidence of renal failure/dialysis in patients.

## Discussion

Aortic arch surgery, recognized as one of the most intricate and high-risk procedures in cardiac surgery, frequently employs HCA as a primary adjunct. The majority of patients undergoing this procedure present with aortic dissection or aneurysm. Compared to other cardiac surgical interventions, aortic arch surgery carries a heightened risk of inadequate perfusion. It is crucial to perform surgical procedures within established safety parameters, particularly in operations involving cerebral ischemia and cerebral protection.^13^ In this meta-analysis, we also added three key time parameters during cardiopulmonary bypass: cardiopulmonary bypass (CPB) time, aortic cross-clamp time, and circulatory arrest time/total cerebral protection time (see Table 5). In the included studies, we observed a reasonable degree of variance in the three time parameters associated with cardiopulmonary bypass, specifically the total cerebral protection time, across the groups. This suggests effective bias control within these studies. Furthermore, the influence of cases with exceptionally prolonged surgical time was mitigated, thereby enhancing the reliability of the findings. The overarching goal of cardiac surgery is to ensure patient safety and minimize surgical time. Recent investigations by cardiac surgeons and perfusionists have focused on perfusion strategies, temperature management, and cannulation methods to reduce inadequate perfusion and improve patient outcomes.

**Table 5:** Statistics of cardiopulmonary bypass-related time parameters in studies

Number	Author	Year	Intervention	Mean (CPB time)	SD (CPB time)	*n* (CPB time)	Mean (aortic cross-clamp time)	SD (aortic cross-clamp time)	*n* (aortic cross-clamp time)	Mean (total cerebral protection time)	SD (total cerebral protection time)	*n* (total cerebral protection time)
1	Huadong Li	2021	MHCA + ACP	208	65.185	36	103	32.593	36	20	8.889	36
			MHCA + BACP	207	77.778	52	114	48.889	52	2	1.4815	52
2	Mark D. Peterson	2020	MHCA + ACP	164.2	68.5	56	134.5	58.3	56	20.4	7.6	56
			MHCA + IA-ACP	179.2	68.3	55	149.1	59.5	55	21.6	9.3	55
3	George Matalanis	2003	DHCA	196	60.4	14	81	67.9	14	25.2	12.4	14
			DHCA + ACP	247.8	86.4	25	137.6	82.1	25	11.4	9.6	25
			DHCA + RCP	193.5	34.9	23	105.8	32.7	23	37.4	19.2	23
4	Christian Olsson	2006	DHCA + ACP	230	42.96	17	–	–	–	33	8.148	17
			MHCA + BACP	276	78.52	17	–	–	–	46	9.63	17
5	Sergey Leontyev	2019	DHCA + ACP	172	59	210	98	36	210	16	8	210
			MHCA + BACP	148	56	210	93	38	210	16	11	210
6	Efstratios Apostolakis	2008	DHCA + ACP	179	28.65	23	–	–	–	39	13.16	23
			DHCA + RCP	184	33.12	25	–	–	–	36	12.73	25
7	YanWenWu	2017	DHCA + ACP	263.4	20.9	50	114	58.4	50	27.4	5.9	50
			MHCA + ACP	207.4	20.9	59	115.9	16.2	59	23.5	6.1	59
8	Richard C. Cook	2006	DHCA + ACP	251	12.6	48	172.4	11.4	48	41	17.04	48
			MHCA + ACP	259.5	15.8	20	180.8	13	20	39	11.85	20
9	Yuji Miyamoto	2003	DHCA	143	62	9	–	–	–	–	–	–
			DHCA + ACP	169	48	14	–	–	–	–	–	–
10	Shuyang Lu	2017	DHCA + ACP	150.7	46.4	135	67.3	30.6	135	34.8	15.1	135
			MHCA + BACP	150.5	49.6	128	73.6	34.7	128	31.8	13.5	128
11	Guang Tong	2017	MHCA + ACP	200	53.75	82	103.5	30.25	82	23	9.25	82
			MHCA + BACP	204	42.5	121	105	31.5	121	24	8	121
12	Yoshiyuki Tokuda	2014	MHCA + ACP	192	54	1,320	116	36	1,320	–	–	–
			MHCA + RCP	174	53	1,320	102	38	1,320	–	–	–
13	Tobias Krüger	2011	DHCA	–	–	–	–	–	–	22.7	14.3	355
			DHCA + ACP	–	––	–	–	–	–	32.2	17.9	628
			MHCA + BACP	–	–	–	–	–	–	–37.6	23.6	453
14	George J. Arnaoutakis	2022	DHCA	177	46.67	133	120	48.89	133	9	3.704	133
			DHCA + RCP	189	50	187	138	55.56	187	15	5.926	187
15	Wenhui Zhang	2025	MHCA + ACP	192	18	76	–	–	–	34.3	4.1	76
			MHCA + BACP	190	20	80	–	–	–	24.6	3.2	80
16	Anna K. Gergen	2023	MHCA + ACP	139	43.7	247	97	36.52	247	10	3.704	247
			MHCA + RCP	128.5	39.04	72	100	26.44	72	7	2.963	72
17	Peiqing Dong	2002	DHCA	204.27	56.78	15	82.47	32.17	15	35.86	18.81	15
			DHCA + RCP	219.72	66.42	50	141.44	48.92	50	45.5	17.21	50
18	Kai Zhu	2022	DHCA + ACP	221.82	56.8	45	128.76	28.07	45	25.69	10.68	45
			MHCA + ACP	182	57.09	43	99.56	39.74	43	24.74	10.31	43
			MHCA + BACP	151.86	30.37	50	77.38	26.09	50	19.12	7.34	50
19	Marco Di Eusanio	2003	DHCA	197	54	128	105	41	128	29	9	128
			MHCA + ACP	198	60	161	129	48	161	42	20	161
20	Oksana Vasilyevna	2017	DHCA	241.3	49.7	29	132.4	55.56	29	51	24.44	29
			MHCA + ACP	213.4	40.67	29	144.7	57.78	29	55	19.26	29
21	Song-Bo Dong	2020	MHCA + ACP	218	39	36	129.3	26.4	36	22.8	4.9	36
			MHCA + BACP	174	29.1	25	96.2	20.4	25	16	4	25
22	A. Faruk Hokenek	2016	MHCA + ACP	155	46.08	15	121.04	38.44	15	23.81	4.85	15
			MHCA + IA-ACP	144.5	49.45	54	112.95	36.72	54	24.69	10.2	54
23	Xiaomeng Wang	2025	DHCA + ACP	204	39.26	599	113	30.37	599	22	8.148	599
			MHCA + ACP	201	39.26	599	112	29.63	599	23	8.148	599
24	Marion Mauduit	2021	DHCA	168.6	64.9	350	120.5	53.8	350	30.3	20	350
			MHCA + ACP	176.6	71.9	74	124.9	53.4	74	38.9	30	74
25	Dominik	2013	DHCA	177	63.5	116	107	35.5	116	36	19	116
			MHCA + ACP	161	50.5	91	109	29.5	91	30	19.5	91
			DHCA + RCP	198	70.75	122	114	42	122	30	18.5	122
26	R. S. Bonser	2002	DHCA	145	24.3	21	96.4	28	21	32	9	21
			DHCA + RCP	153	35.7	21	110.1	39	21	27	12.3	21
27	Wei Wang	2025	DHCA + ACP	199.5	57.9	262	108.5	31	262	22.6	5.3	262
			MHCA + ACP	202.4	74.8	555	127.2	48.1	555	17.5	6.5	555
28	Andreas Zierer	2007	DHCA	200	72	69	129	54	69	36	15	69
			DHCA + RCP	194	65	56	105	50	56	39	15	56
29	Zhongrong Fang	2019	DHCA + ACP	170.6	36.2	217	92.7	21.9	217	20.5	5.7	217
			MHCA + ACP	171.6	45.6	217	98	25.9	217	20.6	6.5	217
30	Yingjie Du	2021	DHCA + ACP	182.3	52.1	191	98.1	26.8	191	21.5	6.8	191
			MHCA + ACP	168.6	48.2	191	97.4	27.4	191	20.8	6.6	191
31	Ming Gong	2016	DHCA + ACP	238	62	35	142	45	35	29	9	35
			MHCA + ACP	211	54	39	118	27	39	28	8	39
32	Mingjia Ma	2016	DHCA + ACP	236.3	35.7	52	128.6	22.7	52	31.5	5.7	52
			MHCA + ACP	218.6	37.1	47	126.5	24.8	47	28	6	47
33	Stevan S. Pupovac	2021	DHCA	195	62.222	362	105	62.222	362	32.5	15.407	362
			MHCA + ACP	186	73.7037	362	106	44.444	362	54	44.444	362
34	Bradley G. Leshnower	2015	DHCA + ACP	214	73	82	141	65	82	37	20	82
			MHCA + ACP	199	74	206	139	64	206	39	20	206
35	Prashanth Vallabhajosyula	2015	DHCA + RCP	222	61	301	163	57	301	23	8	301
			MHCA + IA-ACP	167	49	75	128	46	75	18	5	75
36	Hong Qian	2013	DHCA + ACP	289.5	79.9	33	161.9	34.9	33	44.5	18.4	33
			MHCA + ACP	203.3	61.9	21	130.2	44.6	21	32.1	9.7	21
37	Umberto Benedetto	2021	DHCA	–	–	–	126	69.1	830	33.4	22.2	830
			DHCA + ACP	–	–	–	120	53	117	34.7	20.5	117
			DHCA + RCP	–	–	–	112	53.9	222	31.2	18.5	222
			MHCA + BACP	–	–	–	141	70.4	760	43.7	36	760
38	Andrei M. Belyaev	2024	DHCA + ACP	193.5	44.444	186	121	43.704	186	32	10.37	186
			MHCA + ACP	171	53.333	363	109	41.481	363	29	12.593	363
39	Khaled D. Algarni	2014	DHCA + RCP	174	60	53	89	44	53	29	15	53
			MHCA + RCP	159	71	75	90	59	75	25	13	75
40	Yutaka Okita	2001	DHCA + RCP	175	58	30	99.8	44.3	30	44.3	13.9	30
			MHCA + ACP	213	88	30	103	57	30	54.5	26.2	30
41	Hiroyuki Kamiya	2007	DHCA + ACP	131	44	30	72	25	30	7.5	1.8	30
			MHCA + ACP	156	57	31	94	28	31	9.4	0.8	31
42	James J. Livesay	1983	DHCA	111	5	20	44	4	20	22	2	20
			DHCA + ACP	87	5	40	45	4	40	12	0.9	40
43	Rita Karianna Milewski	2010	RCP	222.9	63.7	682	163.8	59.7	682	25	9.7	682
			MHCA + ACP	171.2	50.3	94	121.3	35.6	94	30.7	7.5	94
44	William B. Keeling	2023	DHCA + RCP	186	82.22	150	138	55.56	150	21	6.667	150
			MHCA + ACP	143	49.63	150	86	44.44	150	20	9.63	150
45	Jacob Ede	2024	DHCA	168	58.52	257	67	41.48	257	19	1.481	257
			DHCA + RCP	202	59.26	258	98	52.59	258	17	2.222	258
46	Sotiris C. Stamou	2018	DHCA	209	108.25	105	–	–	–	31.5	36.5	105
			DHCA + ACP	173.5	43.5	27	–	–	–	18.5	11.5	27
47	W. Brent Keeling	2018	DHCA + ACP	243	78.52	669	142	68.15	669	58	31.11	669
			MHCA + ACP	200	67.41	669	120	55.56	669	63	33.33	669
48	D.K. Harrington	2004	DHCA	221	63.7	21	145	42.4	21	33	9.4	21
			MHCA + ACP	203	41.8	21	152	23.7	21	47	17.7	21
49	Mohammad Shihata	2011	DHCA	200	85	78	127	60	78	38	23	78
			MHCA + ACP	253	58	46	160	57	46	41	19	6
50	Michael E. Halkos	2009	DHCA	218.2	75.9	66	128.9	52.3	66	26	8.5	66
			MHCA + ACP	183.3	64	205	139.1	58.2	205	26.2	12.2	205
51	Hyuk Ahn	1997	DHCA	195.4	55	28	82.3	28.5	28	34.6	17.6	28
			DHCA + ACP	179.8	57.2	12	76.3	19.9	12	30.8	9.3	12
			DHCA + RCP	180.6	30.2	7	88.6	26.2	7	36.3	7.1	7
52	Hend Abdulwahab	2024	DHCA + ACP	255	51.852	103	140	51.852	103	47.6	17.6	103
			MHCA + ACP	210	45.185	40	125	44.444	40	41.3	16	40
53	George J. Arnaoutakis	2016	DHCA + RCP	205	51.85	469	154	52.59	469	22	4.444	469
			MHCA + ACP	178	55.56	117	135	48.89	117	17	4.444	117
54	Bowen Li	2017	DHCA + ACP	187.64	73.02	40	104.36	34.07	40	45.02	16.02	40
			MHCA + BACP	198.71	59.83	37	107.14	36.78	37	44.87	17.33	37
55	Kai Zhang	2024	DHCA + ACP	215	48.33	224	112	28.89	224	24	6.111	224
			MHCA + ACP	165	41.48	433	98	29.63	433	17	4.444	433
56	Hiroyuki Kamiya	2007	DHCA + ACP	289.5	95.8	92	114.6	40.7	92	27.7	16.1	92
			MHCA + ACP	296.1	108.5	92	118.6	42.9	92	28.4	19.9	92
57	Andreas Zierer, MD	2013	MHCA + ACP	164	41	246	99	36	246	41	19	246
			MHCA + BACP	157	37	246	95	41	246	43	21	246
58	Antonio Piperata	2022	MHCA + ACP	170	41.48	189	82	34.07	189	35	11.85	189
			MHCA + BACP	195	52.59	189	100	34.81	189	36	11.85	189
59	Martin Misfeld	2012	DHCA + ACP	186	65	220	98	42	220	15	13	220
			MHCA + RCP	205	60	51	109	41	51	18	12	51
			MHCA +BACP	211	83	242	118	45	242	23	21	242
			MHCA + ACP	208	61	123	110	45	123	22	17	123
60	E lizabeth L. Norton	2022	MHCA + ACP	200	65.19	72	144	53.33	72	28	18.52	72
			MHCA + IA-ACP	222	70.37	192	150	67.41	192	28	13.33	192

This systematic review and network meta-analysis examines non-pharmacological interventions for adverse neurological outcomes related to inadequate perfusion during circulatory arrest, complementing existing meta-analyses in the field with a distinct perspective to enrich the evidence base. In a traditional meta-analysis, the authors compared outcomes between DHCA and MHCA plus selective ACP, finding a significant reduction in stroke incidence associated with MHCA + selective antegrade cerebral perfusion (SACP), while mortality, bleeding, and renal failure showed no significant difference between the groups.^14^ Another meta-analysis included 14 observational studies, focusing on renal outcomes by comparing DHCA (≤20°C) with MHCA (20°C–28°C) regarding postoperative acute kidney injury and dialysis requirement following aortic arch surgery; it concluded that MHCA was associated with significantly lower rates of renal failure and dialysis, particularly when circulatory arrest time was under 30 min.^15^ A third meta-analysis pooled data from 5,060 patients to compare outcomes of ACP and RCP under DHCA conditions, finding no statistically significant difference in 30-day mortality or PND/TND, indicating comparable cerebral protection between the two strategies.^16^ A further meta-analysis demonstrated that DHCA combined with RCP was significantly superior to DHCA alone for aortic arch surgery in reducing mortality and stroke incidence, constituting an optimized cerebral protection strategy, although meta-regression suggested a diminishing benefit of RCP on these outcomes in recent years.^17^ A meta-analysis comparing unilateral versus bilateral ACP found no significant difference in cerebral protection efficacy in aortic arch procedures, as evidenced by no statistically significant differences in short-term mortality, stroke, PND, or TND, or consistent differences in cardiopulmonary bypass or aortic cross-clamp times.^18^ While these meta-analyses incorporated high-quality studies and substantial patient cohorts, each was limited to pairwise comparisons of two interventions, lacking indirect comparisons and precluding comprehensive cross-comparison and ranking of multiple strategies; consequently, they cannot establish a relative effectiveness hierarchy among different approaches. This necessitates high-quality network meta-analyses for supplementation, though few currently exist in this domain. One network meta-analysis investigated four strategies for proximal aortic surgery—DHCA, DHCA + ACP, DHCA + RCP, and MHCA + ACP—finding that MHCA + ACP was optimal for reducing permanent neurological deficit, while DHCA + RCP performed best in lowering perioperative mortality^19^; however, this study encompassed only four strategies and presented conclusions partially inconsistent with the current guidelines. Another network meta-analysis compared the efficacy of cerebral protection strategies in aortic arch surgery, concluding that both ACP and RCP were superior to DHCA alone, with their relative advantage increasing with longer circulatory arrest durations; no significant efficacy difference was found between ACP and RCP.^20^ Nonetheless, this analysis exclusively addressed perfusion strategies without accounting for other influential factors.

Perfusion strategies during DHCA mainly include ACP, RCP, and BACP. The potential mechanisms underlying their effects and limitations are as follows: ACP mimics physiological cerebral perfusion, enabling precise regulation of cerebral blood flow and perfusion pressure to match cerebral metabolic rate of oxygen (CMRO_2_).^21^ By delivering oxygenated blood directly to the brain via the axillary or innominate artery, ACP maintains cerebral oxygen supply-demand balance, thereby extending the DHCA duration and reducing neurological complications by alleviating cerebral ischemia-reperfusion injury (IRI) and oxidative stress. BACP, an optimized version of unilateral ACP, adds the left common carotid artery as an additional perfusion route.^22^^,^^23^ Its core mechanism lies in addressing the vulnerability of the incomplete Willis circle, which may impair collateral circulation during unilateral ACP (typically right sided) and cause contralateral cerebral ischemia/hypoxia.^24^ Even with partial collateral flow, insufficient perfusion may activate apoptotic pathways and neuroinflammation, increasing neurological risk; BACP compensates for this by ensuring bilateral cerebral oxygenation. Clinical studies have shown comparable survival and complication rates between BACP and unilateral ACP,^25^^,^^26^^,^^27^ though the necessity of BACP remains controversial; some studies found no significant neurological adverse events in unilateral ACP patients even with incomplete Willis circle,^28^ while others highlighted BACP’s advantage when the DHCA exceeds 25 min.^24^ RCP’s mechanism involves retrograde perfusion via the superior vena cava to wash out cerebral metabolic by-products, inflammatory mediators, and air emboli, thereby alleviating reperfusion injury, cerebral edema, and embolism risk.^29^^,^^30^^,^^31^ However, its utilization has declined significantly,^32^ as meta-analyses failed to show superiority over ACP in mortality and neurological complications.^33^^,^^34^

Temperature management during HCA is critical. DHCA was once the standard for aortic arch surgery, but MHCA and mild HCA (MI-HCA) hypothermic strategies are increasingly adopted with auxiliary perfusion.^35^ The potential mechanism of temperature optimization is as follows: appropriately increasing nasopharyngeal temperature during arrest avoids excessive cooling-induced organ injury (e.g., kidney and liver) by reducing cold-induced cellular stress and mitochondrial dysfunction,^36^^,^^37^ while maintaining sufficient cerebral protection by balancing CMRO_2_ and oxygen supply. However, some studies still prefer traditional DHCA, as type A dissection patients may benefit more from deep hypothermia via stronger suppression of cerebral metabolism and reduction of IRI.^38^

Cardiopulmonary bypass cannulation methods mainly include axillary, femoral, and innominate artery cannulation and the “branch-first” technique. Axillary artery cannulation, the “gold standard” in many centers,^39^^,^^40^ establishes extracorporeal circulation via prosthetic graft anastomosis; its limitations (brachial plexus injury, infection, etc.) are related to local tissue trauma and perfusion imbalance. Femoral artery cannulation is rapid for emergencies but carries risks of lower extremity ischemia, inadequate cerebral perfusion, and renal dysfunction,^41^^,^^42^^,^^43^ possibly due to retrograde flow-induced microemboli and regional perfusion mismatch. The “branch-first” technique uses a modified trifurcation graft to maintain sequential unilateral-to-bilateral cerebral perfusion during arch replacement,^44^ ensuring continuous cerebral oxygenation and reducing IRI. Our institution prefers combined axillary-femoral cannulation to mitigate single-site risks. Innominate artery cannulation, an alternative to axillary cannulation,^45^ has comparable neurological outcomes,^46^^,^^47^^,^^48^^,^^49^ but its deep location increases dissection-related vascular/nerve injury risk, especially in type A dissection with innominate artery involvement.^50^

This meta-analysis primarily included clinical studies. Due to ethical considerations and feasibility challenges, conducting RCTs was difficult. Therefore, high-quality retrospective cohort studies provided valuable and effective supplementary data. Initially, we assessed the included observational cohort studies and found that most studies effectively controlled for bias risk, such as using propensity score matching to balance baseline patient characteristics. Furthermore, key elements such as patient populations, interventions, and outcome definitions were highly similar between observational cohort studies and randomized clinical trials, significantly reducing selection and confounding biases. Finally, we conducted a sensitivity analysis on four RCTs, using a leave-one-out approach, and found no significant changes in heterogeneity, indicating the stability of the results. Consequently, under these conditions, the combined analysis of data from observational cohort studies and randomized clinical trials is deemed feasible. In addition, given the early adoption of DHCA, we investigated whether the data sources for the DHCA group predated those of other groups. To address this, we reviewed all studies involving the DHCA group alone, totaling 20 publications. Statistical analysis revealed a median publication year of 2010, with the first and third quartiles at 2003 and 2019, respectively. Consequently, the DHCA group data included in this meta-analysis did not significantly differ in publication year compared to the other groups. Furthermore, since DHCA techniques had matured by the 1990s,^51^ the higher proportion of adverse outcomes in the DHCA group cannot be attributed to less advanced surgical, perfusion, or nursing techniques. This suggests no significant confounding bias related to publication year across the groups.

While MHCA did not demonstrate superiority across all outcomes, it remained the most prevalent temperature management strategy.^52^^,^^53^^,^^54^ The findings of this study indicated that MHCA, apart from its uncertain effect on improving paraplegia outcomes, offers significant benefits across other outcome measures. These results aligned with current temperature management strategies, and the safety and substantial advantages of MHCA provided a rationale for further investigation into outcomes related to MI-HCA and even normothermic circulatory arrest.

RCP remains a controversial technique. Some studies suggest that RCP did not increase mortality or postoperative neurological dysfunction and that it was a beneficial adjunct in aortic arch surgery.^55^ However, other research indicated that, under moderate hypothermia, RCP might not demonstrate adequate organ protection compared to ACP.^56^ Based on the results of our meta-analysis, RCP did not demonstrate superior outcomes compared to antegrade or bilateral cerebral perfusion across all outcomes, under identical temperature management and cannulation methods. Consequently, in most scenarios, RCP might not be an optimal strategy for mitigating complications associated with inadequate perfusion.

Regarding innominate artery cannulation, some studies demonstrated its safety and efficacy in aortic arch surgery for cerebral perfusion, ensuring adequate cerebral perfusion.^57^^,^^58^ Innominate artery graft cannulation is a safe and effective method for providing SACP in aortic surgery, particularly in high-risk patient groups such as those with acute aortic dissection and Marfan syndrome. Based on the results of our meta-analysis, antegrade innominate artery perfusion did not demonstrate superiority over other groups in terms of mortality, TND, or PND. Furthermore, it did not show any advantage over moderate hypothermic conditions in terms of renal failure/dialysis and ventilation time. In this meta-analysis, innominate artery showed similar postoperative outcomes to ACP and BACP. The potential causes were that innominate cannulation was established based on the successful cannulation of ACP and as a supplement in certain situations; thus, innominate artery did not appear to have specific advantages. Large-scale, multicenter, and prospective studies are needed to evaluate its long-term efficacy and safety.

Other non-pharmacological interventions are also closely related to HCA, such as pulsatile flow and remote ischemic preconditioning. However, these studies were mostly based on animal experiments with few clinical trials, making them unsuitable for meta-analysis comparison.^59^^,^^60^^,^^61^^,^^62^ As promising non-pharmacological interventions, pulsatile flow and remote ischemic preconditioning are expected to transition from basic research to clinical application in the near future. Furthermore, goal-directed perfusion is a refined and individualized perfusion strategy in cardiopulmonary bypass. It combines oxygen delivery (DO_2_) and oxygen consumption (VO_2_) metabolic indicators, based on routine hemodynamic and oxygen metabolism monitoring, to guide gas and flow management during cardiopulmonary bypass. This intervention is a strategy that aims for optimal perfusion status and usually requires medication. Therefore, it does not strictly meet the criteria for non-pharmacological interventions and is not included in the meta-analysis.

In summary, perfusion strategies, cannulation techniques, and temperature management approaches each present their own advantages and disadvantages. There is no universally optimal combination of interventions, but large-sample analyses can theoretically determine the most effective interventions. Given the variability in patient pathology, cannulation strategies must be tailored to individual patient conditions. Furthermore, the proficiency of the surgical team with specific techniques and their collaborative efforts are of paramount importance. Accordingly, we acknowledge that the interpretation of SUCRA values can at times be overly categorical, as ranking alone does not equate to genuine clinical superiority.

Conclusion: (1) Based on the 60 included studies involving 22,095 patients, compared with profound HCA, moderate hypothermia appears to reduce mortality, the incidence of TND, and the rates of renal failure/dialysis, while also shortening ventilation time. However, it does not significantly impact ICU time or the incidence of paraplegia. (2) RCP cannot improve outcomes related to inadequate perfusion when compared to ACP. (3) Innominate artery cannulation yields similar outcomes to ACP during moderate hypothermia, suggesting that innominate artery cannulation can be used as an alternative to routine ACP. (4) MHCA + BACP and MHCA + ACP are the most recommended intervention strategies. (5) The effect on the incidence of renal failure/dialysis remains uncertain due to identified inconsistencies, which should be interpreted with caution.

### Limitations of the study

The majority of studies included in this article are OCS rather than RCT. Furthermore, the clinical data from these studies originate from various centers. Although we have narrowed the included diseases to aortic dissection and aneurysm, the aforementioned factors inevitably lead to heterogeneity. Studies on great artery transposition, aortic arch interruption, and those related to elderly patients were excluded due to their small sample sizes and diverse patient conditions, which may increase heterogeneity and affect the final results.

The perfusion time, flow rate, and pressure of HCA itself were not discussed in the study. Cerebral perfusion flow rate and perfusion pressure are critical parameters in HCA cerebral perfusion. For ACP, the flow rate is typically 5–10 mL/kg/min, with a pressure maintained at 50–70 mmHg. In contrast, for RCP, the flow rate is usually 100–300 mL/min, with a pressure ≤25 mmHg.^56^^,^^63^ During the study, we observed that numerous studies have investigated the optimal flow rate and perfusion pressure for cerebral perfusion. However, these studies primarily utilized animal models, which, while clinically relevant, were not suitable for inclusion in this study. Furthermore, most clinical centers monitor bilateral cerebral oxygen saturation during HCA. Any abnormalities prompt immediate adjustments to the perfusion flow rate and pressure. Consequently, these parameters are dynamic, precluding statistical analysis and comparative research.

In addition, profound DHCA (PDHCA) is not a routine clinical strategy and MI-HCA is an emerging strategy with limited available studies. Both types of temperature management are associated with limited evidence and high clinical heterogeneity, and thus were not addressed in the present meta-analysis. Accordingly, this study neither performed subgroup analysis on moderate and mild hypothermia temperatures nor did it analyze the effects of shallow hypothermia above 28°C; future research should delve into the impact of different temperature ranges on postoperative outcomes in aortic arch surgery.

Regarding the outcome of renal failure/dialysis, local inconsistency tests showed significant inconsistencies in several comparisons: DHCA + RCP vs. MHCA + RCP (*p* = 0.045), DHCA vs. MHCA (*p* = 0.045), and MHCA vs. MHCA + RCP (*p* = 0.035). The complex structure of the network meta-analysis with multiple closed loops and multi-arm studies may increase the risk of inconsistency. Such inconsistency could reduce the accuracy and reliability of effect estimates for renal failure/dialysis. Therefore, the results related to this outcome should be interpreted with caution.

## Resource availability

All data generated or analyzed during this network meta-analysis are included in this published article and its supplemental information files. The raw datasets, extracted study data, statistical analysis codes, and outcome result files that support the findings of this study are available from the corresponding author upon reasonable request.

### Lead contact

Requests for further information and resources should be directed to and will be fulfilled by the lead contact, Tianxiang Gu (cmugtx@sina.com).

### Materials availability

All raw data and analytical materials supporting this network meta-analysis are provided as supplementary files alongside this manuscript.

### Data and code availability

Data

• This paper analyzes existing, publicly available data, accessible at database (https://doi.org/10.5281/zenodo.20489164).

Code

• All original code is available in this paper’s supplemental information.

Additional information

• Any additional information required to reanalyze the data reported in this paper is available from the lead contact upon request.

## Acknowledgments

We sincerely appreciate all the people who offered help and support during the completion of this study. This work was supported by the following grants: “Hippo signaling pathway inhibition and ferroptosis of neurons targeting circTbcld32: a novel mechanism for improving brain injury by deep hypothermic circulatory arrest” (project number: 2023JH6/100100017) and Research on the application mechanism of “hibernation induction trigger” regulating neuroglobin and its downstream PI3K/Akt and HIF-1α pathways in brain protection during deep hypothermic circulatory arrest (project number: 2023-MSLH-394).

## Author contributions

Conceptualization, T.G. and X.J.; methodology, C.L. and P.Y; investigation, L.M. and Z.W.; writing – original draft, L.M. and Z.W.; writing – review & editing, L.M. and Z.W.; funding acquisition, T.G. and X.J.; resources, Z.Z., E.S., and Q.F.; supervision, T.G. and X.J.

## Declaration of interests

The authors declare no competing interests.

## Declaration of generative AI and AI-assisted technologies in the writing process

In accordance with the stipulations and criteria outlined in the TITAN 2025 guidelines, artificial intelligence was not been utilized in this document.

## STAR★Methods

### Key resources table

**Table:** 

REAGENT or RESOURCE	SOURCE	IDENTIFIER
**Deposited data**		

https://doi.org/10.5281/zenodo.20489164.	N/A	N/A

**Software and algorithms**		

Revman 5.4	N/A	N/A

### Method details

#### Protocol and registration

This systematic review and meta-analysis was conducted in accordance with the Preferred Reporting Items for Systematic Reviews and Meta-Analyses (PRISMA) statement^64^ (http://www.prisma-statement.org/) and was registered on the International Prospective Systematic Review Register (PROSPERO). A completed PRISMA 2020 checklist is provided in supplemental information (Data S2) to document compliance with all reporting items.

#### Information sources and search strategies

The study search encompassed studies published before July 2025, across PubMed, Embase, the Cochrane Library, and Web of Science. The searches were limited to English-language publications and employed a combination of MeSH terms and free-text terms. Search terms included, but were not limited to, circulatory arrest, deep hypothermia induced, deep hypothermic circulatory arrest, antegrade cerebral perfusion, retrograde cerebral perfusion, bilateral cerebral perfusion, moderate hypothermic circulatory arrest, and innominate artery cannulation. The search strategy can be found in the online supplemental information (Data S1).

#### Inclusion and exclusion criteria

The study incorporated nine non-pharmacological interventions: deep hypothermic circulatory arrest (DHCA), deep hypothermic circulatory arrest combined with antegrade cerebral perfusion (DHCA+ACP), deep hypothermic circulatory arrest combined with retrograde cerebral perfusion (DHCA+RCP), deep hypothermic circulatory arrest combined with bilateral antegrade cerebral perfusion (DHCA+BACP), moderate hypothermic circulatory arrest (MHCA), moderate hypothermic circulatory arrest combined with antegrade cerebral perfusion (MHCA+ACP), moderate hypothermic circulatory arrest combined with retrograde cerebral perfusion (MHCA+RCP), moderate hypothermic circulatory arrest combined with bilateral antegrade cerebral perfusion (MHCA+BACP), and moderate hypothermic circulatory arrest combined with innominate artery antegrade cerebral perfusion (MHCA+IA-ACP). Nasopharyngeal temperatures in the DHCA groups ranged from 14.1°C to 20°C, while those in the MHCA groups ranged from 20.1°C to 28°C.^65^ All studies included in the research adhered to the standard temperature range as stipulated by the guidelines. The inclusion criteria were established based on the PICOS principle.^66^ The inclusion criteria for literature screening in this meta-analysis were as follows: (1) Study type: Randomized controlled trial or observational cohort study; (2) Study population: Adult patients undergoing aortic arch surgery for aortic disease; (3) Studies involving at least two or more non-pharmacological interventions; (4) Studies including at least one outcome measure of mortality, permanent neurological dysfunction, or transient neurological dysfunction. The exclusion criteria for literature screening in this meta-analysis were as follows: (1) Unavailable full-text studies; (2) Duplicate studies; (3) Meta-analyses, case reports, abstracts, reviews, conference proceedings, and patents; (4) Studies encompassing only animal experiments and/or cellular assays; (5) Studies with missing or unobtainable key data. (6) Single-arm studies without common control groups. (7)Studies subdividing MHCA into multiple temperature subgroups.

#### Study selection and data acquisition

Two independent reviewers screened the search results. Disagreements during screening were resolved through consultation with a third reviewer to ensure consistency in the screening process. Utilizing EndNote, two independent reviewers merged and de-duplicated the studies. Subsequently, duplicate documents were manually removed. Then screened included studies by reviewing titles and abstracts, and downloaded full texts and rigorously screen according to inclusion and exclusion criteria, and recorded excluded studies and reasons for exclusion. Finally, ascertained the studies for inclusion in the systematic review and created a PRISMA flow diagram. Data extraction induced the following: title, authors, publication year, journal name, country, study type, disease type, intervention method, sample size, age, mortality, permanent neurological dysfunction, transient neurological dysfunction, renal failure/dialysis, paraplegia, ICU tine, and ventilation time. During the data extraction process, no modifications, whether custom or standardized, were applied, and the outcome measures were standardized and aligned with clinical research conventions.

#### Geometry of the network

The network geometry was described in accordance with graph theory criteria.^67^ The reported characteristics included the number of nodes, edges, and studies per edge (represented as edge thickness, with median and interquartile range [IQR]), network density, the proportion of common comparators, and the percentage of strong edges (defined as edges supported by more than one study).

#### Quality assessment and risk of bias assessment

The Newcastle-Ottawa quality assessment scale (NOS) was used to assess the quality of observational cohort studies.^68^ The NOS scale assesses from three angles: study subject selection, group comparability, and exposure factor measurement. When the score is greater than 6, it can be considered a high-quality study. In this meta-analysis, we included OCS studies with a NOS score of ≥6.

The Risk of Bias Assessment Tool I (ROBINS-I) was used to assess the risk of bias of observational cohort studies.^69^ ROBINS-I assesses from aspects such as confounding, classification of interventions, selection of participants into the study, deviations from intended interventions, missing data, measurement of the outcome, and selection of the reported result. Based on the risk level, it is classified as low risk, moderate risk, serious risk, and critical risk.

The Cochrane risk-of-bias tool was used to assess the risk of bias in the included RCT studies. This tool includes seven domains: random sequence generation, allocation concealment, blinding of participants and personnel, blinding of outcome assessment, incomplete outcome data, selective reporting, and other bias. Each domain was rated as low risk, unclear risk, or high risk.^70^

To assess the methodological quality of randomized controlled trials and observational cohort studies, we downloaded and completed the AMSTAR 2 Checklist (https://amstar.ca/Amstar_Checklist.php) and included this form as supplemental information.^71^

The publication bias was evaluated by the Egger’s test, and funnel plots comparing and correcting outcome indicators were generated by visual, with each dot representing an included study. We conducted sensitivity analyses by excluding individual studies sequentially to assess their individual impact on the overall results. The publication bias and sensitivity analyses were conducted with Stata (version 16.0, Stata Corp, College Station, TX, USA). A *p* value < 0.05 indicated statistical significance.

#### Data synthesis and statistics

Given the inherent heterogeneity across studies, a random-effects model was required. We converted the central tendency and dispersion data from the studies into the form of mean and standard deviation^72^ and conducted a network meta-analysis incorporating both full interaction models and random effects for each outcome. We employed mean difference (MD) and 95% confidence intervals (95% CI) to estimate the impact of non-pharmacological interventions on endpoint time, ranking them using P-score, which represents the frequentist equivalent of the surface under the cumulative ranking curve (SUCRA).^73^ To assess the consistency of direct and indirect evidence, we employed node-splitting methods for inconsistency testing, conducting both local and loop inconsistency tests.

Data synthesis was conducted with Review Manager software version 5.4 (Cochrane Collaboration). We reported OR(odds ratio) for dichotomous outcomes and MDs(Mean Difference Score) for continuous outcomes with a 95% CI.

#### Artificial intelligence statement

In accordance with the stipulations and criteria outlined in the TITAN 2025 guidelines,^74^ artificial intelligence was not been utilized in this document.

### Quantification and statistical analysis

We conducted sensitivity analyses by excluding individual studies sequentially to assess their individual impact on the overall results. The publication bias and sensitivity analyses were conducted with Stata (version 16.0, Stata Corp, College Station, TX, USA). A *p* value < 0.05 indicated statistical significance.

### Additional resources

The raw data and supplemental information supporting the findings of this study are publicly available in the Zenodo repository at https://doi.org/10.5281/zenodo.20489164.

## Supplemental information

Document S1. Data S1–S4

Table S1. GRADE certainty grading for pairwise cerebral perfusion comparisons (mortality)

Table S2. GRADE certainty grading for pairwise cerebral perfusion comparisons (PND)

Table S3. GRADE certainty grading for pairwise cerebral perfusion comparisons (TND)

Table S4. GRADE certainty grading for pairwise cerebral perfusion comparisons (renal failure/dialysis)
