# Supplementary material for: Systematic review and network meta-analysis of non-pharmacological interventions for inadequate perfusion in hypothermic circulatory arrest
Source: iScience. 2026 Jul 17;29(8):116722. doi: 10.1016/j.isci.2026.116722 (PMC13400770; doi:10.1016/j.isci.2026.116722)
Supplement: Document S1. Data S1–S4 [file mmc1.pdf]

iScience, Volume *29*

Supplemental information

Systematic review and network meta-analysis of non-pharmacological interventions for inadequate perfusion in hypothermic circulatory arrest

Lingda Meng, Zhijing Wei, Xuan Jiang, Chunmao Lu, Qin Fang, Peng Yu, Zhiwei Zhang, Enyi Shi, and Tianxiang Gu

Data S1 Search Strategy

01-Pubmed

ID	Search	Hits
#1	Search: **"Circulatory Arrest, Deep Hypothermia Induced"[Mesh] OR Circulatory Arrest, Deep Hypothermia Induced [Title/Abstract] OR Deep Hypothermic Circulatory Arrest [Title/Abstract]**	2157
#2	Search: **"antegrade cerebral perfusion"[Title/Abstract] OR "retrograde cerebral perfusion"[Title/Abstract] OR "bilateral cerebral perfusion"[Title/Abstract] OR "distal perfusion"[Title/Abstract] OR "lower body perfusion"[Title/Abstract] OR "moderate hypothermic circulatory arrest"[Title/Abstract] OR "remote ischemic preconditioning"[Title/Abstract] OR "innominate artery cannulation"[Title/Abstract] OR "cerebral oxygen saturation monitoring"[Title/Abstract]**	3087
#3	#1 AND #2	434
		Last Run Date: 07/21/2025

02-WOS

ID	Search	Hits
#1	(TS=("Circulatory Arrest, Deep Hypothermia Induced") OR AB=("Circulatory Arrest, Deep Hypothermia Induced" OR Deep Hypothermic Circulatory Arrest))	2307
#2	(TS=(antegrade cerebral perfusion) OR AB=(antegrade cerebral perfusion))	1535
#3	(TS=(retrograde cerebral perfusion) OR AB=(retrograde cerebral perfusion))	1321
#4	(TS=(bilateral cerebral perfusion) OR AB=(bilateral cerebral perfusion))	2299
#5	(TS=(distal perfusion) OR AB=(distal perfusion))	9114
#6	(TS=(lower body perfusion) OR AB=(lower body perfusion))	8981
#7	(TS=(moderate hypothermic circulatory arrest) OR AB=(moderate hypothermic circulatory arrest))	613
#8	(TS=(remote ischemic preconditioning) OR AB=(remote ischemic preconditioning))	2494
#9	(TS=(innominate artery cannulation) OR AB=(innominate artery cannulation))	261
#10	(TS=(**cerebral oxygen saturation monitoring**) OR AB=(**cerebral oxygen saturation monitoring**))	3238
#11	#2 OR #3 OR #4 OR #5 OR #6 OR #7 OR #8 OR #9 OR #10	27525
#12	#1 AND #11	800
		Last Run Date: 07/21/2025

03-Embase

ID	Search	Hits
#1	'deep hypothermic circulatory arrest'/exp	3021
#2	'hypothermic circulatory arrest':ab,ti	3768
#3	#1 OR #2	5259
#4	'antegrade cerebral perfusion'/exp OR 'retrograde cerebral perfusion'/exp OR 'bilateral cerebral perfusion'/exp OR 'distal perfusion'/exp OR 'lower body perfusion'/exp OR 'moderate hypothermic circulatory arrest'/exp OR ' remote ischemic preconditioning'/exp OR 'innominate artery cannulation'/exp OR 'cerebral oxygen saturation monitoring'/exp	2413
#5	'antegrade cerebral perfusion':ab,ti OR 'retrograde cerebral perfusion':ab,ti OR 'bilateral cerebral perfusion':ab,ti OR 'distal perfusion':ab,ti OR 'lower body perfusion':ab,ti OR 'moderate hypothermic circulatory arrest':ab,ti OR ' remote ischemic preconditioning':ab,ti OR 'innominate artery cannulation':ab,ti OR 'cerebral oxygen saturation monitoring':ab,ti	4452
#6	#4 OR #5	4843
#7	#3 AND #6	993
		Last Run Date: 07/21/2025

04-Cochrane

ID	Search	Hits
#1	MeSH descriptor: [Circulatory Arrest, Deep Hypothermia Induced] explode all trees	33
#2	(Circulatory Arrest, Deep Hypothermia Induced):ti,ab,kw OR(Deep Hypothermic Circulatory Arrest):ti,ab,kw	136
#3	#1 OR #2	136
#4	(antegrade cerebral perfusion OR retrograde cerebral perfusion OR bilateral cerebral perfusion OR distal perfusion OR lower body perfusion OR moderate hypothermic circulatory arrest OR remote ischemic preconditioning OR innominate artery cannulation OR 'cerebral oxygen saturation monitoring):ti,ab,kw	2378
#5	#3 AND #4	42
		Last Run Date: 07/21/2025

Data S2 PRISMA_2020_checklist

PRISMA2020Checklist

Section and Topic	Item #	Checklist item	Location where item is reported
**TITLE**			
Title	1	Systematic review and network meta-analysis of non-pharmacological interventions on inadequate perfusion outcomes in hypothermic circulatory.	Page 1
**ABSTRACT**			
Abstract	2	This article reports the primary research findings according to the abstract checklist, including the inclusion and exclusion criteria, literature sources, and the final search date and risk of bias. The assessment methods are described, increasing the transparency and practicality of the research.	Page 1
**INTRODUCTION**			
Rationale	3	This article provides a detailed description of the research topic and interventions. It also explains the rationale and necessity for conducting a network meta-analysis (NMA). Currently, only a limited number of meta-analyses have compared these interventions, and the included studies are insufficient. This study expands the range of interventions and incorporates a larger number of studies.	Page 1-2
Objectives	4	This article aims to synthesize the impact of non-pharmacological interventions related to hypothermic circulatory arrest on outcomes associated with poor perfusion, clearly defining the research question.	Page 1-2
**METHODS**			
Eligibility criteria	5	This article provides a detailed description of the inclusion and exclusion criteria, encompassing the target population, interventions, control measures, outcome indicators, and study design.	Page 25
Information sources	6	This article encompasses searches of PubMed, Embase, Web of Science, and Cochrane Library databases, with a search timeframe extending from inception through July 2025.	Page 25
Search strategy	7	The search strategies for all databases are provided in Appendix S1.	Page 25
Selection process	8	Initially, two researchers independently screened titles and abstracts, followed by a full-text review based on inclusion and exclusion criteria. Discrepancies were resolved through discussion, with consultation of a third researcher if necessary.	Page 25-26
Data collection process	9	The data extraction process was conducted independently by two researchers using a data extraction form specifically designed for NMA analysis to extract relevant information from all included studies. This included the first author of each RCT, publication year, country where the study was conducted, study design, sample size, gender, age, and outcomes. If data were missing, the researchers contacted the corresponding authors via email to supplement the missing data.	Page 25-26
Data items	10a	The primary outcome measures were mortality, permanent neurological dysfunction, and transient neurological dysfunction. Secondary outcomes included ventilation time, ICU time, paraplegia, and renal failure/dialysis.	Page 3-4
	10b	The authors declare that the funding for this research was utilized for the study, manuscript preparation, and publication of this article. This work was supported by the following grant:<br>"Hippo signaling pathway inhibition and ferroptosis of neurons targeting circTbcld32: A novel mechanism for improving brain injury by deep hypothermic circulatory arrest" (Project Number: 2023JH6/100100017).<br>Research on the application mechanism of "hibernation induction trigger" regulating neuroglobin and its downstream PI3K/Akt and HIF-1α pathways in brain protection during deep hypothermic circulatory arrest (Project Number: 2023-MSLH-394).	Page 10
Study risk of bias assessment	11	Two researchers independently assessed the risk of bias in the included studies using the Cochrane Risk of Bias (ROB) tool. The network geometry was described in accordance with graph theory criteria. The reported characteristics included the number of nodes, edges, and studies per edge (represented as edge thickness, with median and interquartile range [IQR]), network density, the proportion of common comparators, and the percentage of strong edges (defined as edges supported by more than one study). A network graph is provided.	Page 3
Effect measures	12	I calculated the odds ratio and 95% CI using the Mantel-Haenszel method. I assessed global and local inconsistency tests using the Wald test and node-splitting method. SUCRA represents the relative ranking of each intervention for the outcome event.	Page 3
Synthesis	13a	Example Calculating advantages using the Mantel-Haenszel method Ratio and 95% CI. Assessing heterogeneity between studies by determining	Page 3

PRISMA2020Checklist

Section and Topic	Item #	Checklist item	Location where item is reported
methods		I2; e.g. If fruit heterogeneity exists, a random effects model is used for meta-analysis.	
	13b	The entered data is summarized and synthesized.	Page 25
	13c	Use league charts to visualize results.	Page 13
	13d	In this paper, the Mantel-Haenszel method is used to calculate the odds ratio and 95% CI. Heterogeneous analysis between studies was assessed by determining I2; if heterogeneity existed, a random effects model was used for meta-analysis.	Page 3
	13e	This article only discusses the reasons for heterogeneity and does not conduct subgroup analysis or meta-regression.	Page 9-10
	13f	This paper conducted a sensitivity analysis of each outcome indicator.	Page 2
Reporting bias assessment	14	Assess inter-study bias using funnel plots.	Page 2
Certainty assessment	15	Evaluate the certainty of evidence for outcomes using the GRADE approach in Network Meta-Analysis.	Page 5
**RESULTS**			
Study selection	16a	The article search results and screening process are clearly described in the article, with a flowchart providing a visual representation of the complete process from initial search to final study inclusion. Furthermore, the article lists the excluded studies and details the reasons for exclusion for each study, ensuring the transparency and reproducibility of the screening process.	Page 2
	16b	Some studies were excluded after reviewing the full text for various reasons, as detailed in the PRISMA flow diagram.	Page 2
Study characteristics	17	The article cites and references each included study. Furthermore, detailed information for each included study is presented in a table format.	Page 12
Risk of bias in studies	18	The article presents the assessment results for each study across various bias risk domains (e.g., random sequence generation, allocation concealment, blinding, incomplete data, selective reporting) in a clear, graphical format, accompanied by detailed explanations of the assessment results and the rationale behind them for each domain.	Page 2
Results of individual studies	19	The analysis results for each intervention and control group in each study are clearly presented in the form of league tables, and the effect sizes and their 95% CIs are presented in detail to ensure the accuracy of the results.	Page 2
Results of syntheses	20a	This article details the characteristics of the combined results (such as effect sizes, confidence intervals, etc.) and uses funnel plots to represent the risk of bias between studies.	Page 3
	20b	This article shows the combined effect size, precision (such as confidence/confidence interval), and heterogeneity test results for each meta analysis.	Page 3
	20c	This article discusses the reasons for heterogeneity in terms of limitations.	Page 7-8
	20d	No subgroup analysis was performed in this article.	Page 7-8
Reporting biases	21	This paper uses the risk of bias summary diagram and funnel diagram to conduct bias risk analysis.	Page 24
Certainty of evidence	22	Evaluate the certainty of evidence for outcomes using the GRADE approach in Network Meta-Analysis.	Page 3
**DISCUSSION**			
Discussion	23a	This section of the paper provides an in-depth discussion of the statistical findings, offering a detailed explanation of the results.	Page 6-9
	23b	This paper discusses the limitations of the research.	Page 10-11
	23c	No limitations of the review processes used.	Page 6-9

PRISMA2020Checklist

Section and Topic	Item #	Checklist item	Location where item is reported
	23d	This article discusses the impact of statistical results on clinical decision-making.	Page 4-7
**OTHER INFORMATION**			
Registration and protocol	24a	This study protocol has been registered in the International Prospective Systematic Review Register (PROSPERO, CRD42025629887).	Page 25
	24b	Https://www.crd.york.ac.uk/PROSPERO/	Page 25
	24c	No amendments to information provided at registration or in the protocol.	Page 25
Support	25	The authors declare that the funding for this research was utilized for the study, manuscript preparation, and publication of this article. This work was supported by the following grant:<br>"Hippo signaling pathway inhibition and ferroptosis of neurons targeting circTbcld32: A novel mechanism for improving brain injury by deep hypothermic circulatory arrest" (Project Number: 2023JH6/100100017).<br>Research on the application mechanism of "hibernation induction trigger" regulating neuroglobin and its downstream PI3K/Akt and HIF-1α pathways in brain protection during deep hypothermic circulatory arrest (Project Number: 2023-MSLH-394).	Page 10
Competing interests	26	The authors declare that they have no known competing financial interests or personal relationships that could have appeared to influence the work reported in this paper.	Page 10
Availability of data, code and other materials	27	The raw data and supplementary materials supporting the findings of this study are publicly available in the Zenodo repository.	Page 10

From: Page MJ, McKenzie JE, Bossuyt PM, Boutron I, Hoffmann TC, Mulrow CD, et al. The PRISMA 2020 statement: an updated guideline for reporting systematic reviews. BMJ 2021;372:n71. doi: 10.1136/bmj.n71. This work is licensed under CC BY 4.0. To view a copy of this license, visit https://creativecommons.org/licenses/by/4.0/

Data S3 Other supplemental figures

1.mortality_RoB_chart

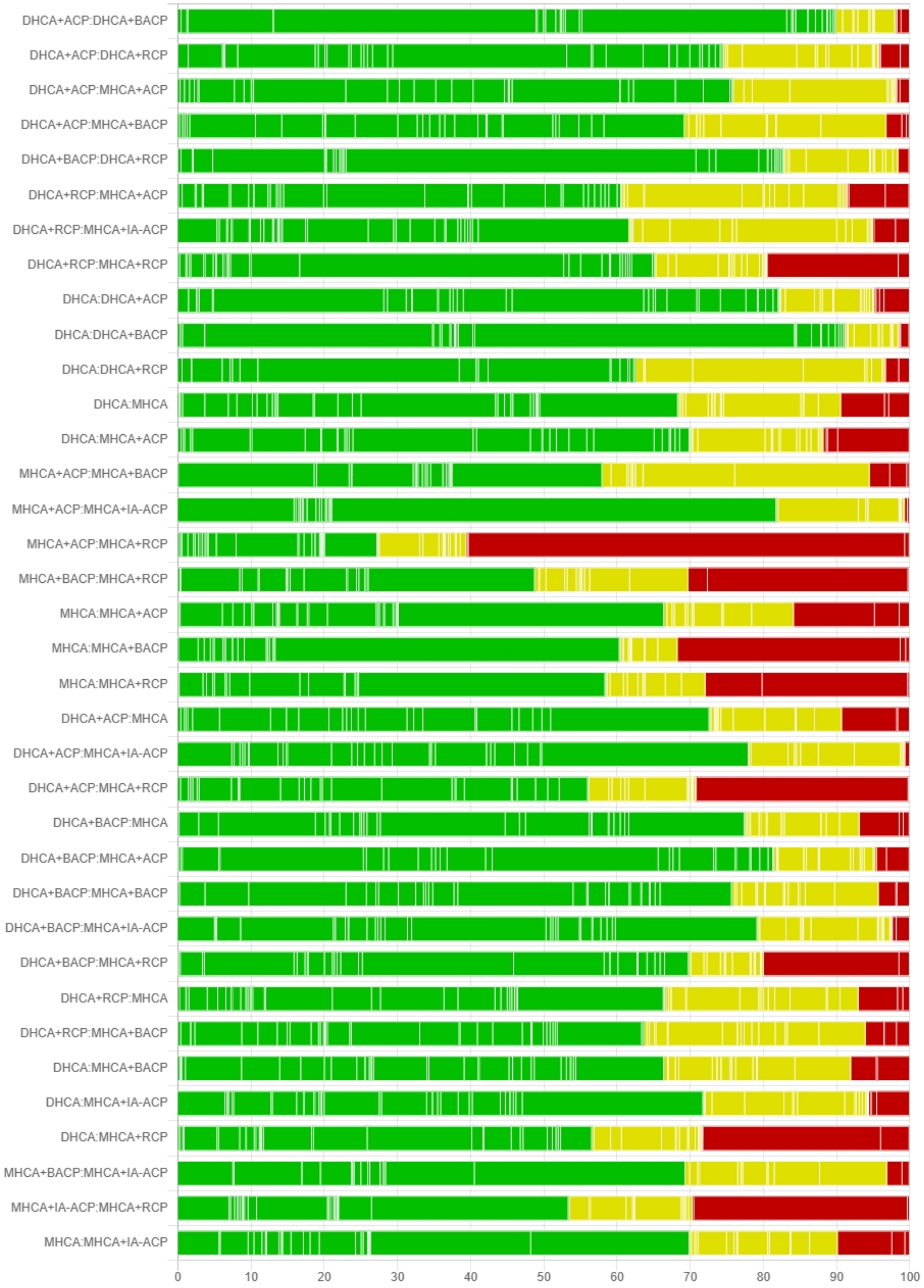

2.PND_RoB_chart

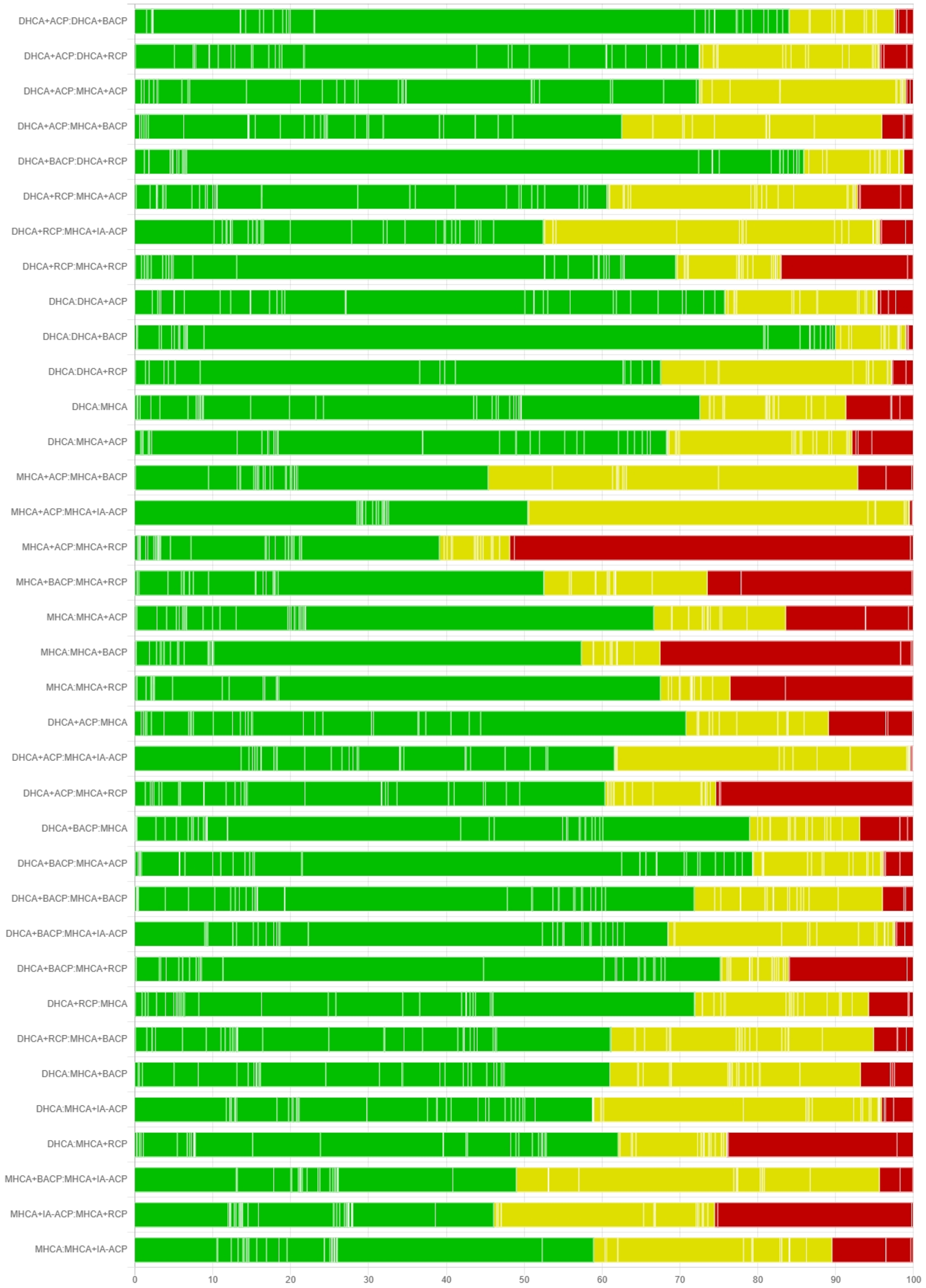

3.TND_RoB_chart

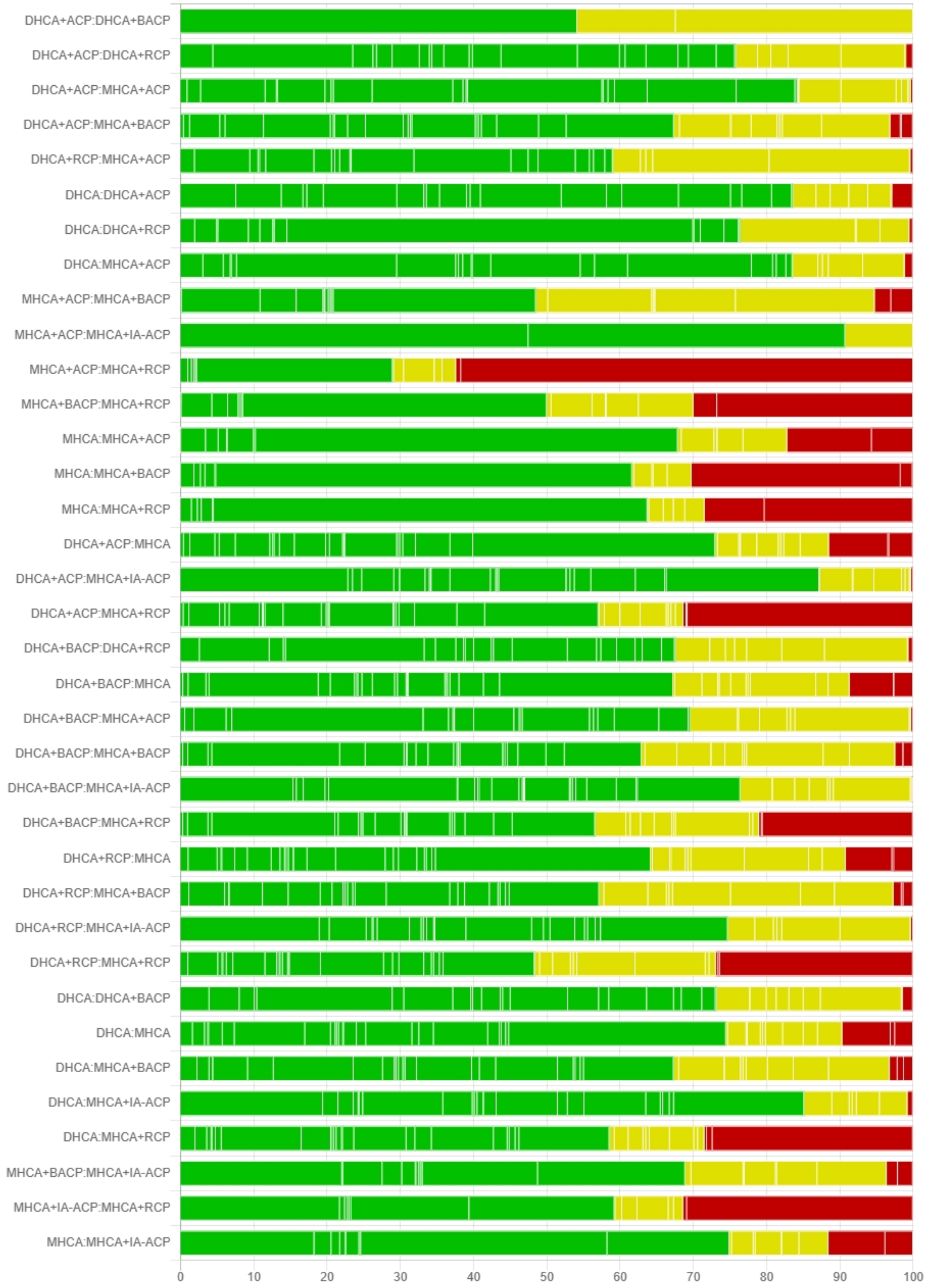

4.renal failure_RoB_chart

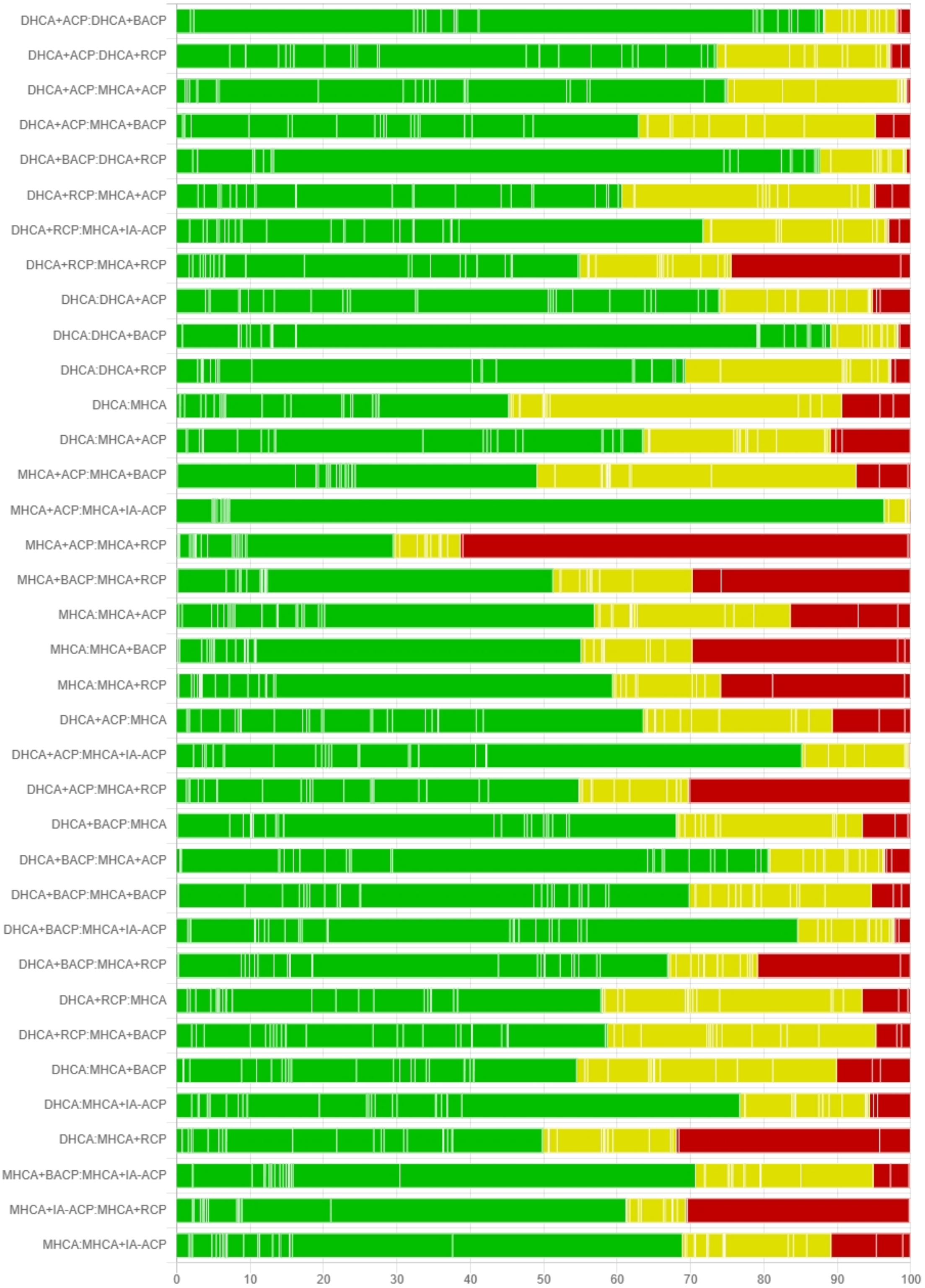

Data S4 global test of inconsistency

PND： The global test of inconsistency using the design-by-treatment interaction model showed no significant inconsistency for PND ($\chi^2 = 73.416$, df = 58, $P = 0.084$), supporting the consistency assumption of the network meta-analysis.

TND： The global test of inconsistency using the design-by-treatment interaction model showed no significant inconsistency for TND ($\chi^2 = 49.136$, df = 40, $P = 0.152$), supporting the consistency assumption of the network meta-analysis.

Renal Failure / Dialysis,： The global test of inconsistency using a random-effects design-by-treatment interaction modelrevealed $\chi^2 = 65.580$ (df = 47), $P = 0.038$,indicating significant global inconsistency. The consistency assumption of the network meta-analysis was violated.

Mortality：The global test of inconsistency using the design-by-treatment interaction model showed no significant inconsistency for mortality ($\chi^2 = 79.591$, df = 61, $P = 0.055$), supporting the consistency assumption of the network meta-analysis.

Incoherence

Define clinically important size of effect: Risk ratio 1 Set Reset

*Relative effect estimates below **1.000** and above **1.000** are considered clinically important.*
Importance of Incoherence depends on the variability of direct and indirect effects in relation to a clinically important size of effect

Global test based on a random-effects design-by-treatment interaction model

χ² statistic: 73.416 (58 degrees of freedom), P value: 0.084

Local tests: Separating indirect from direct evidence

Reset Proceed

Comparison	Evidence	NMA risk ratio	Direct risk ratio	Indirect risk ratio	Inconsistency measures: Ratio of risk ratios	P value	Incoherence judgment
DHCA:DHCA+ACP	mixed	1.501(1.063,2.118)	0.652(0.337,1.260)	0.672(0.449,1.007)	0.970(0.447,2.102)	0.938	No concerns
DHCA:DHCA+BACP	mixed	1.352(0.854,2.140)	1.027(0.599,1.761)	0.310(0.129,0.746)	3.311(1.183,9.265)	0.023	Major concerns
DHCA:DHCA+RCP	mixed	1.440(1.058,1.960)	0.714(0.505,1.010)	0.622(0.316,1.224)	1.148(0.537,2.455)	0.722	No concerns
DHCA:MHCA	mixed	1.215(0.712,2.074)	0.411(0.134,1.257)	1.011(0.550,1.858)	0.406(0.114,1.452)	0.166	No concerns
DHCA:MHCA+ACP	mixed	1.739(1.295,2.334)	0.563(0.385,0.824)	0.593(0.373,0.944)	0.950(0.521,1.732)	0.867	No concerns
DHCA+ACP:DHCA+BACP	mixed	0.901(0.551,1.474)	1.107(0.587,2.087)	0.659(0.302,1.438)	1.680(0.615,4.592)	0.312	No concerns
DHCA+ACP:DHCA+RCP	mixed	0.960(0.656,1.404)	0.902(0.446,1.823)	0.985(0.626,1.548)	0.916(0.397,2.114)	0.837	No concerns
DHCA+ACP:MHCA+ACP	mixed	1.158(0.907,1.480)	1.067(0.817,1.396)	1.754(0.959,3.207)	0.609(0.315,1.178)	0.140	No concerns
DHCA+ACP:MHCA+BACP	mixed	1.395(0.892,2.184)	4.445(0.923,21.411)	1.260(0.790,2.010)	3.528(0.684,18.191)	0.132	No concerns
DHCA+BACP:DHCA+RCP	mixed	1.065(0.657,1.728)	1.118(0.605,2.068)	0.984(0.449,2.157)	1.136(0.419,3.077)	0.802	No concerns
DHCA+RCP:MHCA+ACP	mixed	1.207(0.861,1.693)	0.878(0.489,1.577)	1.416(0.936,2.143)	0.620(0.303,1.270)	0.192	No concerns
DHCA+RCP:MHCA+IA-ACP	mixed	2.080(0.788,5.488)	4.257(0.239,75.967)	1.898(0.677,5.319)	2.243(0.105,47.851)	0.605	No concerns
DHCA+RCP:MHCA+RCP	mixed	1.193(0.749,1.900)	1.981(0.945,4.155)	0.856(0.470,1.558)	2.314(0.893,5.997)	0.084	No concerns
MHCA:MHCA+ACP	mixed	1.431(0.878,2.332)	1.186(0.559,2.518)	1.640(0.863,3.114)	0.723(0.269,1.945)	0.521	No concerns
MHCA:MHCA+BACP	mixed	1.724(1.075,2.764)	2.390(1.400,4.079)	0.543(0.199,1.485)	4.398(1.408,13.736)	0.011	Major concerns
MHCA:MHCA+RCP	mixed	1.414(0.792,2.525)	0.898(0.379,2.129)	2.054(0.939,4.495)	0.437(0.136,1.402)	0.164	No concerns
MHCA+ACP:MHCA+BACP	mixed	1.205(0.818,1.773)	0.885(0.580,1.349)	5.924(2.270,15.462)	0.149(0.052,0.426)	0.000	Major concerns
MHCA+ACP:MHCA+IA-ACP	mixed	1.723(0.685,4.333)	1.588(0.600,4.200)	3.562(0.196,64.851)	0.446(0.021,9.513)	0.605	No concerns
MHCA+ACP:MHCA+RCP	mixed	0.988(0.652,1.497)	0.842(0.512,1.384)	1.433(0.672,3.056)	0.587(0.237,1.454)	0.250	No concerns
MHCA+BACP:MHCA+RCP	mixed	0.820(0.486,1.386)	0.527(0.214,1.299)	1.028(0.540,1.957)	0.513(0.169,1.553)	0.237	No concerns

Comparison	Evidence	Indirect risk ratio	Inconsistency measures	Incoherence judgment
DHCA:MHCA+BACP	indirect	0.477(0.299,0.763)	Not applicable	Some concerns
DHCA:MHCA+IA-ACP	indirect	0.334(0.128,0.874)	Not applicable	Some concerns
DHCA:MHCA+RCP	indirect	0.582(0.363,0.934)	Not applicable	Some concerns
DHCA+ACP:MHCA	indirect	0.810(0.473,1.385)	Not applicable	Some concerns
DHCA+ACP:MHCA+IA-ACP	indirect	1.996(0.770,5.175)	Not applicable	Some concerns
DHCA+ACP:MHCA+RCP	indirect	1.145(0.714,1.835)	Not applicable	Some concerns
DHCA+BACP:MHCA	indirect	0.899(0.461,1.752)	Not applicable	Some concerns
DHCA+BACP:MHCA+ACP	indirect	1.286(0.790,2.093)	Not applicable	Some concerns
DHCA+BACP:MHCA+BACP	indirect	1.549(0.842,2.851)	Not applicable	Some concerns
DHCA+BACP:MHCA+IA-ACP	indirect	2.216(0.785,6.250)	Not applicable	Some concerns
DHCA+BACP:MHCA+RCP	indirect	1.270(0.690,2.338)	Not applicable	Some concerns
DHCA+RCP:MHCA	indirect	0.844(0.480,1.484)	Not applicable	Some concerns
DHCA+RCP:MHCA+BACP	indirect	1.454(0.885,2.390)	Not applicable	Some concerns
MHCA:MHCA+IA-ACP	indirect	2.466(0.870,6.988)	Not applicable	Some concerns
MHCA+BACP:MHCA+IA-ACP	indirect	1.430(0.527,3.885)	Not applicable	Some concerns
MHCA+IA-ACP:MHCA+RCP	indirect	0.573(0.209,1.571)	Not applicable	Some concerns

Incoherence

Define clinically important size of effect: Risk ratio 1 Set Reset

*Relative effect estimates below **1.000** and above **1.000** are considered clinically important.*
Importance of Incoherence depends on the variability of direct and indirect effects in relation to a clinically important size of effect

Global test based on a random-effects design-by-treatment interaction model

χ² statistic: 49.136 (40 degrees of freedom), P value: 0.152

Local tests: Separating indirect from direct evidence

Reset Proceed

Comparison DHCA:DHCA+ACP
Evidence: mixed
NMA risk ratio: 1.061(0.654,1.721)
Direct risk ratio: 1.576(0.489,5.079)
Indirect risk ratio: 0.848(0.499,1.441)
Inconsistency measures
Ratio of risk ratios: 1.859(0.514,6.719)
P value: 0.344
Incoherence judgment: No concerns

Comparison DHCA:DHCA+RCP
Evidence: mixed
NMA risk ratio: 1.322(0.882,1.981)
Direct risk ratio: 0.618(0.389,0.983)
Indirect risk ratio: 1.439(0.629,3.296)
Inconsistency measures
Ratio of risk ratios: 0.430(0.166,1.110)
P value: 0.081
Incoherence judgment: Major concerns

Comparison DHCA:MHCA+ACP
Evidence: mixed
NMA risk ratio: 1.464(0.936,2.290)
Direct risk ratio: 0.851(0.456,1.589)
Indirect risk ratio: 0.542(0.285,1.029)
Inconsistency measures
Ratio of risk ratios: 1.570(0.641,3.843)
P value: 0.324
Incoherence judgment: No concerns

Comparison DHCA+ACP:DHCA+BACP
Evidence: direct
Direct risk ratio: 2.621(1.233,5.572)
Inconsistency measures: Not applicable
Incoherence judgment: No concerns

Comparison DHCA+ACP:DHCA+RCP
Evidence: mixed
NMA risk ratio: 1.246(0.769,2.020)
Direct risk ratio: 0.786(0.298,2.073)
Indirect risk ratio: 1.451(0.831,2.533)
Inconsistency measures
Ratio of risk ratios: 0.541(0.177,1.657)
P value: 0.282
Incoherence judgment: No concerns

Comparison DHCA+ACP:MHCA+ACP
Evidence: mixed
NMA risk ratio: 1.379(1.065,1.786)
Direct risk ratio: 1.389(1.059,1.822)
Indirect risk ratio: 1.284(0.546,3.020)
Inconsistency measures
Ratio of risk ratios: 1.082(0.441,2.653)
P value: 0.864
Incoherence judgment: No concerns

Comparison DHCA+ACP:MHCA+BACP
Evidence: mixed
NMA risk ratio: 1.978(1.321,2.962)
Direct risk ratio: 2.593(0.663,10.145)
Indirect risk ratio: 1.927(1.263,2.941)
Inconsistency measures
Ratio of risk ratios: 1.345(0.323,5.613)
P value: 0.684
Incoherence judgment: No concerns

Comparison DHCA+RCP:MHCA+ACP
Evidence: mixed
NMA risk ratio: 1.107(0.705,1.738)
Direct risk ratio: 1.276(0.644,2.529)
Indirect risk ratio: 0.991(0.543,1.809)
Inconsistency measures
Ratio of risk ratios: 1.287(0.518,3.200)
P value: 0.587
Incoherence judgment: No concerns

Comparison MHCA:MHCA+ACP
Evidence: mixed
NMA risk ratio: 0.885(0.541,1.449)
Direct risk ratio: 0.770(0.402,1.476)
Indirect risk ratio: 1.067(0.502,2.270)
Inconsistency measures
Ratio of risk ratios: 0.722(0.266,1.955)
P value: 0.521
Incoherence judgment: No concerns

Comparison MHCA:MHCA+BACP
Evidence: mixed
NMA risk ratio: 1.269(0.797,2.022)
Direct risk ratio: 1.240(0.751,2.047)
Indirect risk ratio: 1.469(0.416,5.187)
Inconsistency measures
Ratio of risk ratios: 0.844(0.217,3.282)
P value: 0.807
Incoherence judgment: No concerns

Comparison MHCA:MHCA+RCP
Evidence: mixed
NMA risk ratio: 0.962(0.527,1.758)
Direct risk ratio: 0.721(0.319,1.631)
Indirect risk ratio: 1.361(0.557,3.325)
Inconsistency measures
Ratio of risk ratios: 0.530(0.158,1.778)
P value: 0.304
Incoherence judgment: No concerns

Comparison MHCA+ACP:MHCA+BACP
Evidence: mixed
NMA risk ratio: 1.434(1.045,1.968)
Direct risk ratio: 1.388(0.998,1.930)
Indirect risk ratio: 2.096(0.678,6.473)
Inconsistency measures
Ratio of risk ratios: 0.662(0.205,2.145)
P value: 0.492
Incoherence judgment: No concerns

Comparison MHCA+ACP:MHCA+IA-ACP
Evidence: direct
Direct risk ratio: 1.233(0.468,3.249)
Inconsistency measures: Not applicable
Incoherence judgment: No concerns

Comparison MHCA+ACP:MHCA+RCP
Evidence: mixed
NMA risk ratio: 1.087(0.700,1.690)
Direct risk ratio: 1.097(0.697,1.726)
Indirect risk ratio: 0.930(0.138,6.283)
Inconsistency measures
Ratio of risk ratios: 1.179(0.166,8.398)
P value: 0.869
Incoherence judgment: No concerns

Comparison MHCA+BACP:MHCA+RCP
Evidence: mixed
NMA risk ratio: 0.758(0.459,1.254)
Direct risk ratio: 0.843(0.380,1.872)
Indirect risk ratio: 0.707(0.370,1.351)
Inconsistency measures
Ratio of risk ratios: 1.192(0.427,3.329)
P value: 0.738
Incoherence judgment: No concerns

Comparison DHCA:DHCA+BACP
Evidence: indirect
Indirect risk ratio: 0.360(0.147,0.880)
Inconsistency measures: Not applicable
Incoherence judgment: No concerns

Comparison DHCA:MHCA
Evidence: indirect
Indirect risk ratio: 0.605(0.311,1.176)
Inconsistency measures: Not applicable
Incoherence judgment: No concerns

Comparison DHCA:MHCA+BACP
Evidence: indirect
Indirect risk ratio: 0.476(0.276,0.824)
Inconsistency measures: Not applicable
Incoherence judgment: No concerns

Comparison DHCA:MHCA+IA-ACP
Evidence: indirect
Indirect risk ratio: 0.554(0.191,1.611)
Inconsistency measures: Not applicable
Incoherence judgment: No concerns

Comparison DHCA:MHCA+RCP
Evidence: indirect
Indirect risk ratio: 0.628(0.335,1.178)
Inconsistency measures: Not applicable
Incoherence judgment: No concerns

Comparison DHCA+ACP:MHCA
Evidence: indirect
Indirect risk ratio: 1.558(0.895,2.713)
Inconsistency measures: Not applicable
Incoherence judgment: No concerns

Comparison DHCA+ACP:MHCA+IA-ACP
Evidence: indirect
Indirect risk ratio: 1.701(0.624,4.635)
Inconsistency measures: Not applicable
Incoherence judgment: No concerns

Comparison DHCA+ACP:MHCA+RCP
Evidence: indirect
Indirect risk ratio: 1.500(0.900,2.499)
Inconsistency measures: Not applicable
Incoherence judgment: No concerns

Comparison DHCA+BACP:DHCA+RCP
Evidence: indirect
Indirect risk ratio: 0.475(0.194,1.164)
Inconsistency measures: Not applicable
Incoherence judgment: No concerns

Comparison DHCA+BACP:MHCA
Evidence: indirect
Indirect risk ratio: 0.595(0.233,1.516)
Inconsistency measures: Not applicable
Incoherence judgment: No concerns

Comparison DHCA+BACP:MHCA+ACP
Evidence: indirect
Indirect risk ratio: 0.526(0.237,1.168)
Inconsistency measures: Not applicable
Incoherence judgment: No concerns

Comparison DHCA+BACP:MHCA+BACP
Evidence: indirect
Indirect risk ratio: 0.755(0.321,1.775)
Inconsistency measures: Not applicable
Incoherence judgment: No concerns

Comparison DHCA+BACP:MHCA+IA-ACP
Evidence: indirect
Indirect risk ratio: 0.649(0.185,2.275)
Inconsistency measures: Not applicable
Incoherence judgment: No concerns

Comparison DHCA+BACP:MHCA+RCP
Evidence: indirect
Indirect risk ratio: 0.572(0.230,1.422)
Inconsistency measures: Not applicable
Incoherence judgment: No concerns

Comparison DHCA+RCP:MHCA
Evidence: indirect
Indirect risk ratio: 1.251(0.641,2.439)
Inconsistency measures: Not applicable
Incoherence judgment: No concerns

Comparison DHCA+RCP:MHCA+BACP
Evidence: indirect
Indirect risk ratio: 1.587(0.915,2.752)
Inconsistency measures: Not applicable
Incoherence judgment: No concerns

Comparison DHCA+RCP:MHCA+IA-ACP
Evidence: indirect
Indirect risk ratio: 1.365(0.469,3.974)
Inconsistency measures: Not applicable
Incoherence judgment: No concerns

Comparison DHCA+RCP:MHCA+RCP
Evidence: indirect
Indirect risk ratio: 1.204(0.640,2.262)
Inconsistency measures: Not applicable
Incoherence judgment: No concerns

Comparison MHCA:MHCA+IA-ACP
Evidence: indirect
Indirect risk ratio: 1.091(0.368,3.236)
Inconsistency measures: Not applicable
Incoherence judgment: No concerns

Comparison MHCA+BACP:MHCA+IA-ACP
Evidence: indirect
Indirect risk ratio: 0.860(0.310,2.383)
Inconsistency measures: Not applicable
Incoherence judgment: No concerns

Comparison MHCA+IA-ACP:MHCA+RCP
Evidence: indirect
Indirect risk ratio: 0.882(0.304,2.557)
Inconsistency measures: Not applicable
Incoherence judgment: No concerns

Incoherence

Define clinically important size of effect: Risk ratio 1 Set Reset

*Relative effect estimates below **1.000** and above **1.000** are considered clinically important.*

Importance of Incoherence depends on the variability of direct and indirect effects in relation to a clinically important size of effect

Global test based on a random-effects design-by-treatment interaction model

χ² statistic: 65.580 (47 degrees of freedom), P value: 0.038

Local tests: Separating indirect from direct evidence

Reset Proceed

Comparison	Evidence	NMA risk ratio	Direct risk ratio	Indirect risk ratio	Inconsistency measures: Ratio of risk ratios	P value	Incoherence judgment
DHCA:DHCA+ACP	mixed	0.784(0.596,1.030)	0.960(0.504,1.827)	1.359(1.005,1.837)	0.706(0.347,1.438)	0.338	No concerns
DHCA:DHCA+BACP	mixed	0.730(0.503,1.059)	1.490(0.933,2.379)	1.186(0.643,2.188)	1.256(0.581,2.715)	0.562	No concerns
DHCA:DHCA+RCP	mixed	0.954(0.712,1.277)	1.011(0.720,1.420)	1.162(0.657,2.055)	0.870(0.448,1.688)	0.680	No concerns
DHCA:MHCA	mixed	1.114(0.739,1.679)	1.489(0.783,2.829)	0.634(0.372,1.080)	2.349(1.020,5.410)	0.045	No concerns
DHCA:MHCA+ACP	mixed	1.085(0.861,1.367)	0.870(0.655,1.157)	1.031(0.694,1.532)	0.844(0.518,1.374)	0.494	No concerns
DHCA+ACP:DHCA+BACP	mixed	0.931(0.639,1.357)	0.809(0.503,1.301)	1.180(0.638,2.183)	0.686(0.315,1.492)	0.342	No concerns
DHCA+ACP:DHCA+RCP	mixed	1.217(0.880,1.684)	0.903(0.482,1.694)	1.356(0.928,1.982)	0.666(0.319,1.388)	0.278	No concerns
DHCA+ACP:MHCA+ACP	mixed	1.384(1.153,1.661)	1.384(1.139,1.682)	1.383(0.822,2.328)	1.001(0.574,1.744)	0.999	No concerns
DHCA+ACP:MHCA+BACP	mixed	1.727(1.211,2.463)	4.445(0.945,20.909)	1.639(1.139,2.360)	2.711(0.553,13.307)	0.219	No concerns
DHCA+BACP:DHCA+RCP	mixed	1.307(0.875,1.952)	1.125(0.664,1.909)	1.601(0.864,2.967)	0.703(0.312,1.583)	0.395	No concerns
DHCA+RCP:MHCA+ACP	mixed	1.137(0.845,1.531)	0.916(0.576,1.458)	1.320(0.898,1.942)	0.694(0.379,1.269)	0.236	No concerns
DHCA+RCP:MHCA+IA-ACP	mixed	2.331(1.089,4.990)	3.255(0.180,58.751)	2.274(1.033,5.005)	1.431(0.071,28.714)	0.815	No concerns
DHCA+RCP:MHCA+RCP	mixed	1.165(0.749,1.813)	3.538(1.093,11.452)	0.970(0.602,1.563)	3.646(1.026,12.958)	0.045	Major concerns
MHCA:MHCA+ACP	mixed	0.974(0.658,1.441)	0.954(0.481,1.892)	0.983(0.610,1.586)	0.970(0.421,2.236)	0.943	No concerns
MHCA:MHCA+BACP	mixed	1.215(0.821,1.799)	0.988(0.625,1.563)	2.140(1.002,4.571)	0.462(0.190,1.120)	0.087	Major concerns
MHCA:MHCA+RCP	mixed	0.998(0.616,1.617)	0.560(0.273,1.151)	1.596(0.833,3.058)	0.351(0.133,0.926)	0.035	No concerns
MHCA+ACP:MHCA+BACP	mixed	1.248(0.916,1.701)	1.242(0.885,1.744)	1.278(0.599,2.725)	0.972(0.424,2.229)	0.947	No concerns
MHCA+ACP:MHCA+IA-ACP	mixed	2.050(1.010,4.162)	2.007(0.967,4.165)	2.873(0.157,52.647)	0.699(0.035,14.012)	0.815	No concerns
MHCA+ACP:MHCA+RCP	mixed	1.025(0.718,1.462)	0.941(0.639,1.385)	1.622(0.661,3.984)	0.580(0.218,1.543)	0.275	No concerns
MHCA+BACP:MHCA+RCP	mixed	0.821(0.532,1.266)	0.615(0.304,1.245)	0.978(0.565,1.693)	0.628(0.257,1.536)	0.308	No concerns
DHCA:MHCA+BACP	indirect			0.739(0.511,1.069)	Not applicable		Major concerns
DHCA:MHCA+IA-ACP	indirect			0.450(0.214,0.945)	Not applicable		Major concerns
DHCA:MHCA+RCP	indirect			0.900(0.597,1.357)	Not applicable		Major concerns
DHCA+ACP:MHCA	indirect			1.422(0.928,2.177)	Not applicable		Major concerns
DHCA+ACP:MHCA+IA-ACP	indirect			2.837(1.366,5.892)	Not applicable		Major concerns
DHCA+ACP:MHCA+RCP	indirect			1.418(0.954,2.108)	Not applicable		Major concerns
DHCA+BACP:MHCA	indirect			1.526(0.904,2.576)	Not applicable		Major concerns
DHCA+BACP:MHCA+ACP	indirect			1.486(1.019,2.167)	Not applicable		Major concerns
DHCA+BACP:MHCA+BACP	indirect			1.854(1.148,2.996)	Not applicable		Major concerns
DHCA+BACP:MHCA+IA-ACP	indirect			3.046(1.369,6.779)	Not applicable		Major concerns
DHCA+BACP:MHCA+RCP	indirect			1.523(0.915,2.533)	Not applicable		Major concerns
DHCA+RCP:MHCA	indirect			1.168(0.732,1.865)	Not applicable		Major concerns
DHCA+RCP:MHCA+BACP	indirect			1.419(0.934,2.157)	Not applicable		Major concerns
MHCA:MHCA+IA-ACP	indirect			1.996(0.889,4.480)	Not applicable		Major concerns
MHCA+BACP:MHCA+IA-ACP	indirect			1.643(0.759,3.557)	Not applicable		Major concerns
MHCA+IA-ACP:MHCA+RCP	indirect			0.500(0.226,1.103)	Not applicable		Major concerns

Incoherence

Define clinically important size of effect: Risk ratio 1 Set Reset

*Relative effect estimates below **1.000** and above **1.000** are considered clinically important.*

Importance of Incoherence depends on the variability of direct and indirect effects in relation to a clinically important size of effect

Global test based on a random-effects design-by-treatment interaction model

χ² statistic: 79.591 (61 degrees of freedom), P value: 0.055

Local tests: Separating indirect from direct evidence

Reset Proceed

Comparison	Evidence	NMA risk ratio	Direct risk ratio	Indirect risk ratio	Inconsistency measures: Ratio of risk ratios	P value	Incoherence judgment
DHCA:DHCA+ACP	mixed	1.073(0.852,1.350)	0.823(0.584,1.160)	1.031(0.757,1.406)	0.798(0.503,1.267)	0.340	No concerns
DHCA:DHCA+BACP	mixed	1.054(0.793,1.401)	0.947(0.683,1.314)	0.952(0.534,1.697)	0.995(0.512,1.932)	0.988	No concerns
DHCA:DHCA+RCP	mixed	1.003(0.785,1.283)	1.017(0.770,1.342)	0.926(0.544,1.576)	1.098(0.603,2.001)	0.759	No concerns
DHCA:MHCA	mixed	1.614(1.008,2.583)	0.265(0.110,0.635)	0.875(0.501,1.529)	0.302(0.107,0.854)	0.024	Major concerns
DHCA:MHCA+ACP	mixed	1.315(1.062,1.627)	0.843(0.633,1.123)	0.670(0.487,0.921)	1.258(0.819,1.931)	0.294	No concerns
DHCA+ACP:DHCA+BACP	mixed	0.983(0.737,1.312)	0.944(0.682,1.307)	1.140(0.609,2.132)	0.829(0.409,1.679)	0.602	No concerns
DHCA+ACP:DHCA+RCP	mixed	0.935(0.700,1.249)	0.964(0.556,1.672)	0.925(0.658,1.299)	1.042(0.545,1.991)	0.901	No concerns
DHCA+ACP:MHCA+ACP	mixed	1.226(1.020,1.473)	1.267(1.029,1.560)	1.092(0.740,1.612)	1.160(0.746,1.804)	0.510	No concerns
DHCA+ACP:MHCA+BACP	mixed	1.291(0.885,1.882)	1.111(0.225,5.481)	1.302(0.883,1.920)	0.853(0.165,4.409)	0.850	No concerns
DHCA+BACP:DHCA+RCP	mixed	0.952(0.683,1.327)	0.917(0.560,1.502)	0.981(0.626,1.538)	0.934(0.480,1.821)	0.842	No concerns
DHCA+RCP:MHCA+ACP	mixed	1.310(0.995,1.725)	1.053(0.608,1.825)	1.410(1.026,1.937)	0.747(0.396,1.410)	0.368	No concerns
DHCA+RCP:MHCA+IA-ACP	mixed	0.968(0.427,2.194)	0.997(0.109,9.088)	0.963(0.399,2.324)	1.035(0.096,11.176)	0.978	No concerns
DHCA+RCP:MHCA+RCP	mixed	1.616(1.082,2.415)	1.857(0.952,3.625)	1.495(0.904,2.470)	1.243(0.538,2.868)	0.611	No concerns
MHCA:MHCA+ACP	mixed	0.815(0.520,1.276)	1.498(0.680,3.299)	0.609(0.353,1.051)	2.458(0.941,6.418)	0.066	No concerns
MHCA:MHCA+BACP	mixed	0.858(0.565,1.302)	0.946(0.591,1.515)	0.600(0.244,1.476)	1.577(0.571,4.353)	0.380	No concerns
MHCA:MHCA+RCP	mixed	1.005(0.582,1.736)	1.449(0.491,4.272)	0.886(0.470,1.670)	1.634(0.467,5.723)	0.442	No concerns
MHCA+ACP:MHCA+BACP	mixed	1.053(0.752,1.475)	0.959(0.665,1.383)	1.768(0.748,4.180)	0.542(0.213,1.381)	0.200	No concerns
MHCA+ACP:MHCA+IA-ACP	mixed	0.738(0.337,1.616)	0.735(0.319,1.697)	0.761(0.082,7.060)	0.966(0.089,10.435)	0.978	No concerns
MHCA+ACP:MHCA+RCP	mixed	1.234(0.863,1.764)	1.077(0.710,1.634)	1.805(0.898,3.629)	0.597(0.265,1.346)	0.213	No concerns
MHCA+BACP:MHCA+RCP	mixed	1.171(0.732,1.874)	1.791(0.619,5.186)	1.057(0.626,1.784)	1.695(0.518,5.544)	0.383	No concerns

Comparison	Evidence	Indirect risk ratio	Inconsistency measures	Incoherence judgment
DHCA:MHCA+BACP	indirect	0.722(0.491,1.062)	Not applicable	Some concerns
DHCA:MHCA+IA-ACP	indirect	1.030(0.460,2.308)	Not applicable	Some concerns
DHCA:MHCA+RCP	indirect	0.617(0.416,0.914)	Not applicable	Some concerns
DHCA+ACP:MHCA	indirect	1.504(0.935,2.421)	Not applicable	Some concerns
DHCA+ACP:MHCA+IA-ACP	indirect	0.905(0.406,2.019)	Not applicable	Some concerns
DHCA+ACP:MHCA+RCP	indirect	1.512(1.021,2.239)	Not applicable	Some concerns
DHCA+BACP:MHCA	indirect	1.531(0.904,2.592)	Not applicable	Some concerns
DHCA+BACP:MHCA+ACP	indirect	1.247(0.918,1.694)	Not applicable	Some concerns
DHCA+BACP:MHCA+BACP	indirect	1.313(0.840,2.053)	Not applicable	Some concerns
DHCA+BACP:MHCA+IA-ACP	indirect	0.921(0.399,2.125)	Not applicable	Some concerns
DHCA+BACP:MHCA+RCP	indirect	1.538(0.979,2.418)	Not applicable	Some concerns
DHCA+RCP:MHCA	indirect	1.608(0.969,2.670)	Not applicable	Some concerns
DHCA+RCP:MHCA+BACP	indirect	1.380(0.903,2.109)	Not applicable	Some concerns
MHCA:MHCA+IA-ACP	indirect	0.602(0.244,1.482)	Not applicable	Some concerns
MHCA+BACP:MHCA+IA-ACP	indirect	0.701(0.299,1.644)	Not applicable	Some concerns
MHCA+IA-ACP:MHCA+RCP	indirect	1.670(0.708,3.939)	Not applicable	Some concerns
